# pH-Responsive Erdafitinib Liposomes with FGFR3 Targeting for Intravesical Bladder Cancer Therapy

**DOI:** 10.3390/ijms27209014

**Published:** 2026-10-11

**Authors:** Hongxia Liang, Galong Li, Wenli Zhang, Wei Wang, Dongyan Li, Yuanyuan Cheng, Minghui Song, Xiaopan Xu, Hongbing Lu

**Affiliations:** 1 School of Biomedical Engineering, Fourth Military Medical University, Xi’an 710032, China; 18549824697@163.com (H.L.); ligalong@fmmu.edu.cn (G.L.); wenli1121@fmmu.edu.cn (W.Z.); 18326832462@163.com (D.L.); chengyuanyuan0319@163.com (Y.C.); 15653811835@163.com (M.S.); 2 Medical Innovation Center, Fourth Military Medical University, Xi’an 710032, China; 3 Department of Pharmaceutics and Pharmacy Administration, School of Pharmacy, Fourth Military Medical University, Xi’an 710032, China; wangwei_fmmu@163.com

**Keywords:** nanoliposomes, pH-responsive drug delivery, bladder cancer, erdafitinib, *FGFR3*-targeted therapy, intravesical administration

## Abstract

Fibroblast growth factor receptor 3 (*FGFR3*) is a key oncogenic driver in bladder cancer (BCa). Although erdafitinib, the pioneering *FGFR3* inhibitor, is approved for clinic use, its oral administration frequently causes inadequate therapeutic effect and side effects. Here, we report a pH-responsive and erdafitinib-loaded liposomal nanosystem with surface modification of anti-*FGFR3* antibodies (Phl-erda@Ab), which demonstrates improved antitumor effects in intravesical instillation therapy. In RT112 cells, we evaluated targeted uptake and antiproliferative activity. In vivo, we assessed intravesical retention, antitumor efficacy, and histopathological markers (TUNEL, Ki67) in a BCa xenograft *BALB/C nude mouse* model, alongside systemic safety profiling. The Phl-erda@Ab exhibited sharp pH sensitivity, releasing 49% of the drug payload within 1 h at pH 5.3 while maintaining minimal release at pH 7.4 and 6.5. Liposome retention after intravesical instillation was doubled compared with non-targeted Phl-erda in mice. The Phl-erda@Ab intravesical therapy demonstrated effective antitumor activity, including maximal necrosis, the highest TUNEL-positive apoptotic index, and the lowest Ki67 proliferation index, compared to other groups. No significant pathological damage to major organs was observed. In summary, Phl-erda@Ab enables *FGFR3*-targeted, pH-triggered intravesical delivery with enhanced intravesical retention, improved antitumor activity, and favorable local safety, providing preclinical evidence to support clinical translation in *FGFR3*-overexpressing BCa.

## 1. Introduction

Urinary bladder cancer (BCa) represents the sixth most common cancer among men worldwide, and the leading malignancy in the urinary system [1,2]. According to the latest global cancer statistics in 2024, about 614,000 new BCa cases were annually diagnosed, with an estimated 221,000 deaths globally [3]. In clinical practice, BCa is dichotomized into non-muscle-invasive (NMIBC, ~70–75% of new cases) and muscle-invasive (MIBC) subtypes, each carrying markedly divergent prognostic trajectories [4,5].

Intravesical instillation therapy, recommended by clinical guidelines for the management of both NMIBC and MIBC, offers a minimally invasive procedure that preserves bladder function and limits systemic toxicity [6,7,8]. In practice, NMIBC patients routinely receive intravesical chemotherapy or Bacillus Calmette-Guérin immunotherapy after transurethral resection to reduce recurrence [9,10,11,12]. For MIBC patients who are unfit for radical cystectomy, intravesical instillation is also incorporated into bladder-preserving regimens [13,14,15]. In both settings, however, the lack of specific tumor-targeting capability, compounded by spontaneous bladder voiding that limits drug residence time and concentration, has consistently confined this therapy to the adjuvant setting. Hence, developing novel intravesical instillation therapies with enhanced tumor targeting, fewer side effects, and good efficacy remains a desirable need in BCa management.

Fibroblast growth factor receptor 3 (*FGFR3*) is a well-established oncogenic driver in BCa [16,17]. Its activating mutations or overexpression fuel tumor proliferation, suppress apoptosis, and promote angiogenesis, all of which processes are inextricably linked to disease progression and poor clinical outcomes [18,19,20]. *FGFR3* alterations occur across the entire BCa stages, from NMIBC to MIBC, highlighting its universal relevance as a druggable target [17,21,22,23].

While erdafitinib has obtained FDA approval as the pioneering *FGFR3* inhibitor for patients with chemotherapy-unresponsive BCa, its oral administration frequently provokes hyperphosphatemia and stomatitis, which often result in dose reduction or therapeutic interruption, severely narrowing the treatment window [16,24,25,26]. Considering the hollow-organ anatomical structure of the bladder lumen, intravesical administration offers a feasible way to maximize local drug exposure while minimizing systemic absorption [14,27]. However, whether such localized delivery can overcome bladder microenvironment barriers to achieve tumor-selective and therapeutically effective exposure remains an open key question. Liposomes nanocarriers offer biocompatibility, mucoadhesion, and receptor-mediated endocytosis that bypasses efflux resistance [28,29]. pH-sensitive linkages (e.g., imide bonds) enable stable intravesical drug retention at physiological/urine pH but rapid release in acidic tumor microenvironment (pH ≤ 5.3). This spatiotemporal control minimizes premature drug loss during instillation while maximizing bioavailability at the tumor site. Furthermore, surface conjugation of anti-*FGFR3* antibodies converts passive carriers into active targeting vectors, enabling precise tumor recognition and amplifying drug internalization through antibody–antigen binding [30,31].

**Figure 1:**
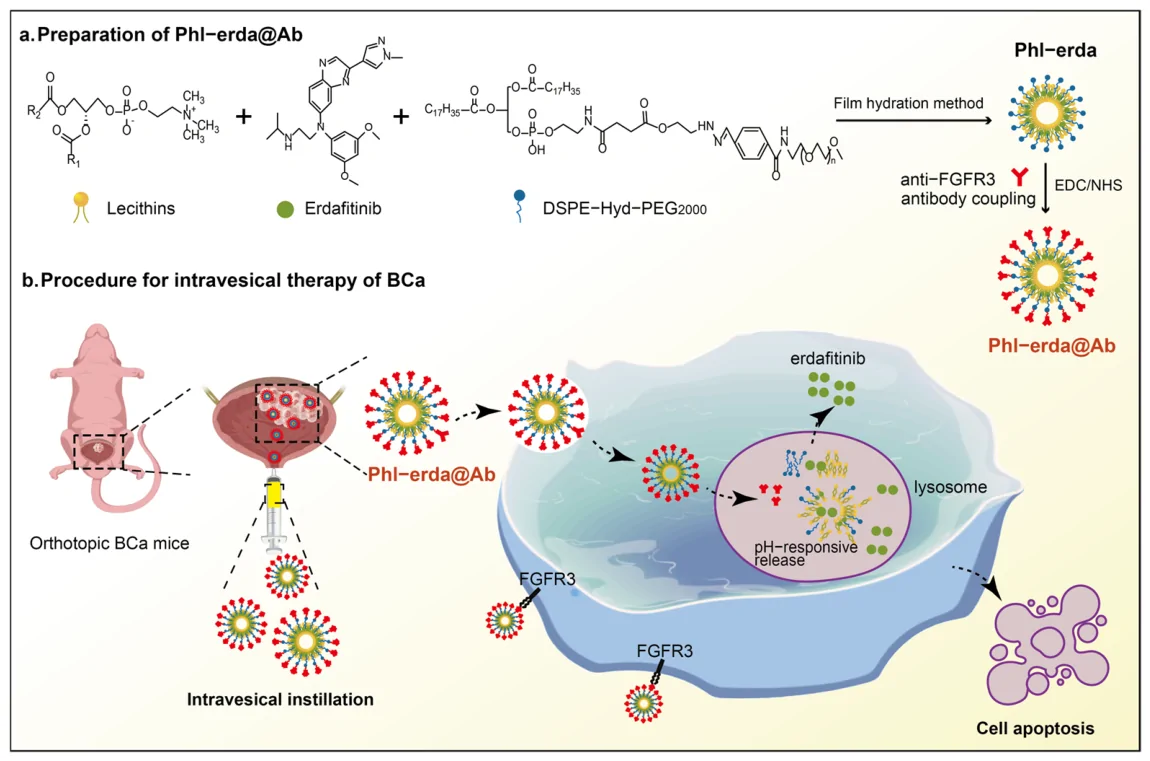
Schematic illustration of the fabrication of pH-responsive, erdafitinib-loaded, anti-*FGFR3* antibody-conjugated liposomes (Ph-erda@Ab) and their intravesical therapy workflow for *FGFR3*-overexpressing bladder cancer.

Herein, we developed a pH-responsive, *FGFR3*-targeted, erdafitinib-loaded antibody-liposomal nanosystem (Phl-erda@Ab) for intravesical therapy of *FGFR3*-positive BCa. (Figure 1). This nanosystem integrates active targeting, mucosal retention, and triggered drug release. Phl-erda@Ab exhibited sharp pH sensitivity, releasing the drug rapidly at acidic pH (5.3) but minimally at neutral or urine pH. Compared with non-targeted controls, it doubled intravesical drug retention, significantly reduced tumor fluorescence, and induced maximal apoptosis with minimal proliferation. No major organ toxicity was observed. Thus, Phl-erda@Ab provides a targeted, pH-gated intravesical nanotherapy with enhanced intravesical retention and potent antitumor activity, supporting clinical translation in *FGFR3*-overexpressing BCa.

## 2. Results

### 2.1. Characterization of Liposome Drug Delivery System

To prepare erdafitinib-loaded, anti-*FGFR3* antibody-conjugated pH-responsive liposomes (denoted as Phl-erda@Ab), a pH-responsive liposome (Phl) was first fabricated using the thin-film hydration method. Subsequently, erdafitinib was loaded into Phl via the same method to yield Phl-erda. Finally, Phl-erda@Ab was obtained by conjugating anti-*FGFR3* antibodies onto the surface of Phl-erda.

**Figure 2:**
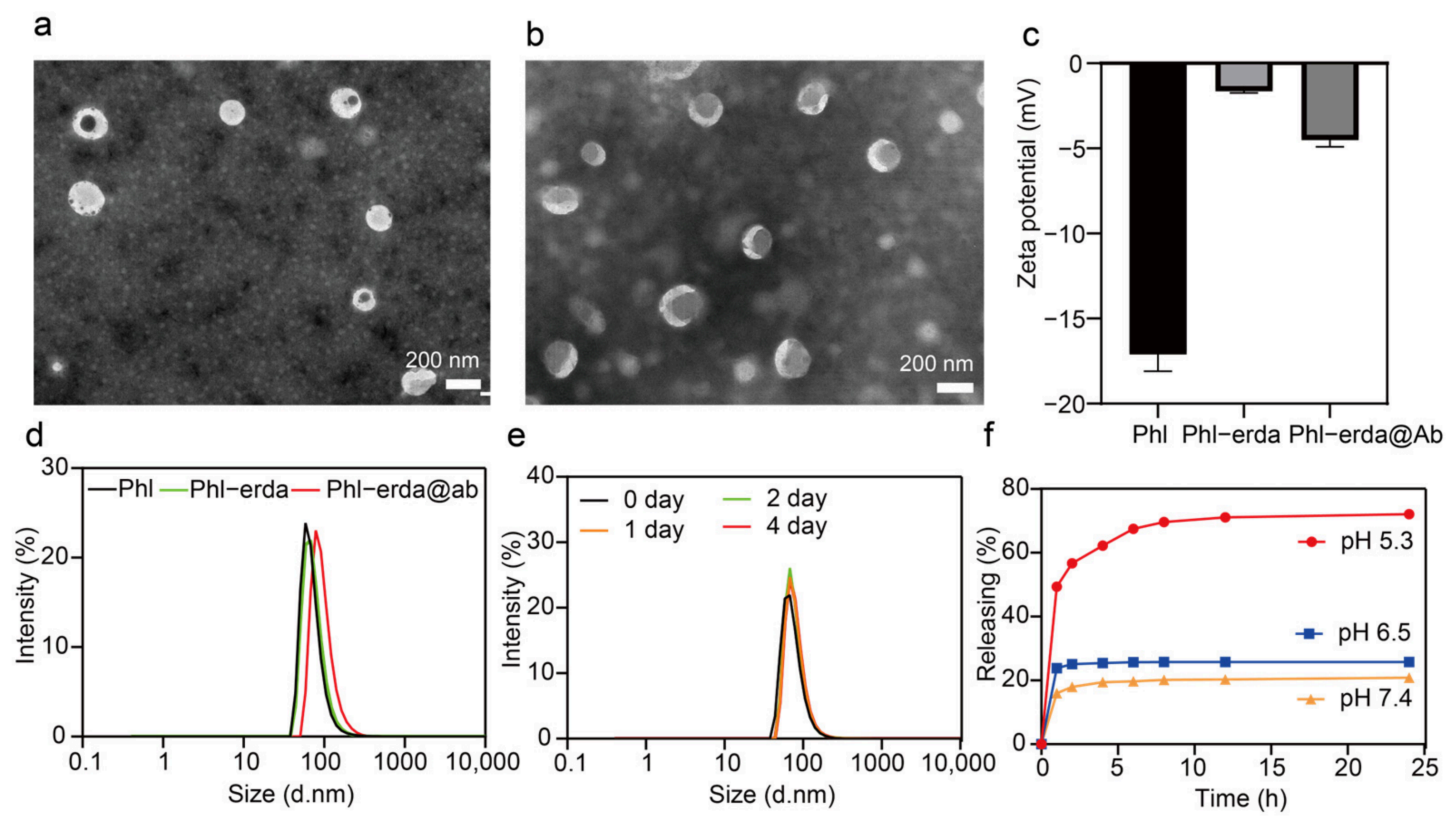
Characterization of Phl-erda. Negatively stained TEM image of (**a**) Phl and (**b**) Phl-erda. Scale bar: 200 nm. (**c**) Zeta potential of Phl, Phl-erda, and Phl-erda@Ab. (**d**) The hydrodynamic diameters of Phl, Phl-erda, and Phl-erda@Ab were 58.77 nm, 68.06 nm, and 78.82 nm, with corresponding PDI values of 0.191, 0.225, and 0.131, respectively. (**e**) The hydrodynamic diameters of Phl-erda on days 0, 1, 2, and 4 after synthesis were 68.06 nm, 68.06 nm, 68.06 nm, and 68.10 nm, with corresponding PDI values of 0.225, 0.510, 0.374, and 0.361, respectively. (**f**) Drug release profiles of Phl-erda at different pH values.

The antibody conjugation amount and conjugation efficiency of Phl-erda@Ab were determined by the BCA assay at 570 nm using a microplate reader. Based on the calibration curve of BSA protein standards (Figure S4) and the calculation formula, the amount of coupling antibody of Phl-erda@Ab was found to be 265 µg Ab/μmol PL, with a coupling efficiency of 71.60%. The morphologies of Phl and Phl-erda were characterized by TEM. As the TEM image shown in Figure 2a,b, Phl-erda showed an average particle size of approximately 78 nm, which was significantly larger than 24 nm of Phl. The hydrodynamic size of the samples was further measured by DLS. The hydrodynamic diameters of Phl, Phl-erda, and Phl-erda@Ab were 58.77 nm, 68.06 nm, and 78.82 nm, respectively (Figure 2d). And the zeta potentials of Phl, Phl-erda, and Phl-erda@Ab were Phl-erda@Ab −17 mV, −2 mV, and −5 mV, respectively (Figure 2c). Since the drug was ultimately intended for intravesical instillation therapy in this study, the zeta potentials and hydrodynamic diameters of the formulations in artificial urine were further determined. As shown in Figure S5a, the zeta potentials of Phl, Phl-erda, and Phl-erda@Ab were −5.93 mV, −1.77 mV, and −3.16 mV, respectively. The hydrodynamic diameters of Phl, Phl-erda, and Phl-erda@Ab were 274.1 nm, 231.6 nm, and 209.6 nm, respectively (Figure S5b). In addition, the stability of Phl-erda in artificial urine was evaluated by DLS on days 1, 4, and 8, and the corresponding hydrodynamic diameters were 231.6 nm, 221.6 nm, and 325 nm, respectively (Figure S5c). As determined by LC-MS, the drug (erdafitinib) loading content was 76.96%. These data collectively demonstrated that erdafitinib was efficiently encapsulated within the liposomes, and the anti-*FGFR3* antibodies were successfully modified on the surface of Phl-erda.

The colloidal stability of Phl-erda@Ab in water was assessed by tracking its particle size and polydispersity index (PDI) continuously for 4 days. The hydrodynamic diameter of Phl-erda@Ab showed no significant change throughout the 4-day observation period (Figure 2e), and the PDI of Phl-erda remained within an acceptable range, indicating that Phl-erda possessed good stability in water.

The pH-responsive Phl-erda showed different erdafitinib release behaviors in solutions at different pH levels. The released erdafitinib from Phl was collected by dialysis method and determined by LC-MS. The Phl-erda-containing PBS solutions for dialysis at pH = 7.4, pH = 6.5, and pH = 5.3 were used to simulate the pH of blood, tumor microenvironment, and lysosomal microenvironment. After LC-MS measurements, the released amount of erdafitinib was obtained and used for calculating the drug release rate according to formula (2.2). As shown in Figure 2f, the $Q_{t}$ value of erdafitinib were 21% and 26% over 24 h in PBS solutions at pH 7.4 and pH 6.5, respectively. Importantly, the $Q_{t}$ of erdafitinib reached up to 72% within 24 h, indicating a high drug release in the acidic lysosomal microenvironment of BCa cells. This phenomenon was attributed to the hydrolysis of the hydrazone bond in the liposome under acidic conditions. These results demonstrated that Phl-erda possesses a pronounced pH-responsive release profile, highlighting its potential for superior drug release behavior within acid tumor microenvironment.

### 2.2. In Vitro Experiments

#### 2.2.1. Targeting Ability of Phl-erda@Ab

**Figure 3:**
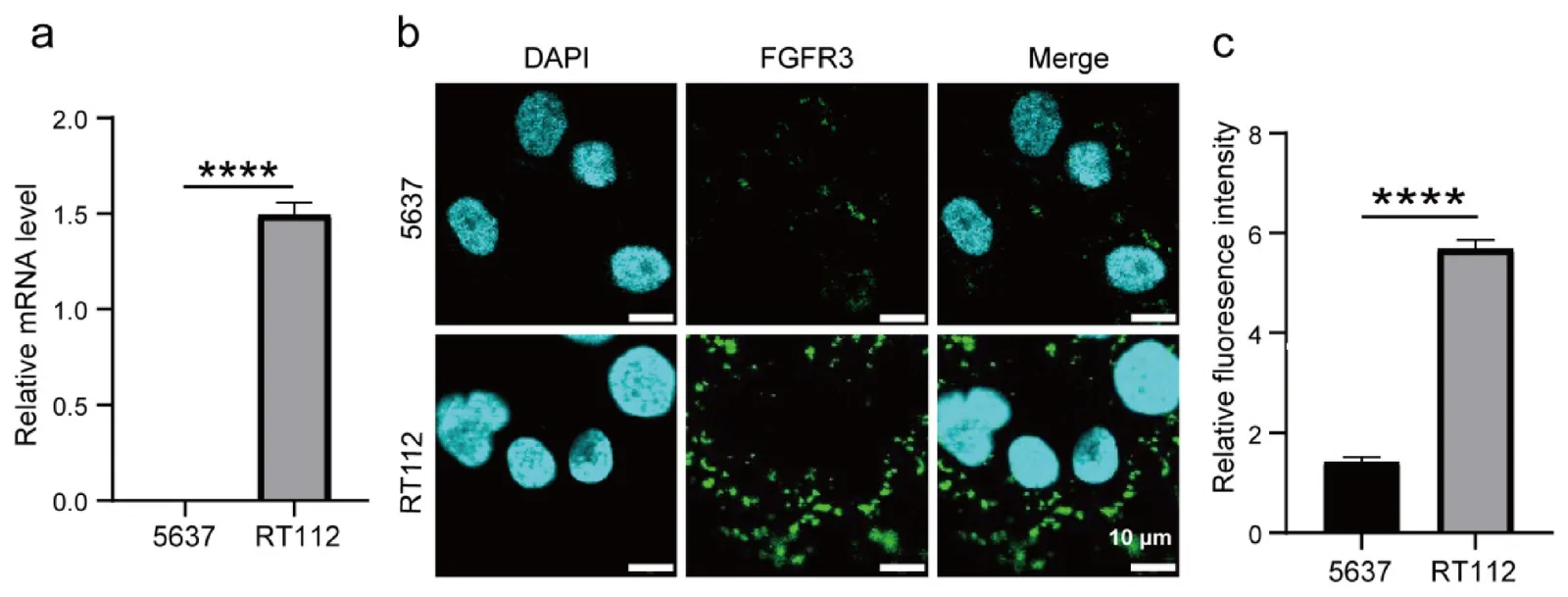
In vitro targeting ability of Phl-erda@Ab to FGFR3-overexpressing RT11 cells. (**a**) Relative mRNA expression levels of *FGFR3* in RT112 and 5637 cells. (**b**) CLSM images of RT112 and 5637 cells after 1 h of co-incubation with FITC (green)-labeled Phl-erda@Ab. Cell nuclei were stained with DAPI (blue). Scale bar: 10 µm. (**c**) Quantitative analysis of green fluorescence intensity of Phl-erda@Ab from confocal images. Data were analyzed using a two-tailed Student’s *t*-test (*n* = 3). **** *p* < 0.0001.

An in vitro experiment was performed to validate the ability of Phl-erda@Ab to target *FGFR3*-overexpressing RT112 cells. First, the mRNA expression levels of *FGFR3* in RT112 were evaluated by qPCR. As shown in Figure 3a, the relative mRNA expression level of *FGFR3* in RT112 cells was significantly higher than that in 5637 cells. The expression levels of *FGFR3* protein in RT112, RT4, and 5637 cells were detected by Western blotting (WB). As shown in Figure S9, the *FGFR3* protein level in RT112 cells was significantly higher than that in 5637 cells. Although the *FGFR3* protein level in RT112 cells was higher than that in RT4 cells, the two levels were relatively comparable. Therefore, RT112 cells with high *FGFR3* expression were used for subsequent in vitro and in vivo investigations, while 5637 cells were used as a control. Based on this, Phl-erda@Ab was surface modified with FITC fluorescein, after which the FITC-labeled Phl-erda@Ab was incubated with RT112 cells and 5637 cells for 1 h, respectively.

Confocal laser scanning microscopy (CLSM) was used to evaluate the targeting ability of Phl-erda@Ab. As presented in the CLSM images in Figure 3b,c, an obvious green fluorescence signal of FITC-labeled Phl-erda@Ab can be observed around RT112 cells, whereas the green fluorescence signal was significantly weaker around 5637 cells (Figure 3b). Quantitative analysis indicated that the mean fluorescence intensity in the RT112 group was 3.99 times higher than that in the 5637 group. These results demonstrated that Phl-erda@Ab can specifically and efficiently bind to *FGFR3*-overexpressing RT112 cells, which is beneficial for the precise therapy of BCa in vitro and in vivo.

#### 2.2.2. Cellular Uptake of Phl-erda@Ab

The superior cellular uptake of Phl-erda@Ab is a key step to induce apoptosis in cancer cells. To investigate whether the cellular uptake efficiency of Phl-erda@Ab depends on its *FGFR3* antibody targeting, we performed a receptor blockade experiment using a non-blockade group as the control. Phl-erda@Ab was labeled with Cy5.5 and incubated with cells for 1 h, followed by imaging and quantitative analysis by confocal laser scanning microscopy and flow cytometry. As shown in Figure S6a,b, drug uptake was comparable between RT112 and RT4 cells, whereas it was relatively lower in 5637 cells. Cellular uptake in the blockade group was generally lower than that in the non-blockade group and the cell uptake in the non-blocking group is about 1.5 times that of the blocking group, with a statistically significant difference. Flow cytometry results (Figure S6c,d) showed a consistent trend. The non-blockade group and the cell uptake in the non-blocking group is about 1.3 times that of the blocking group. These results indicate that blocking *FGFR3* receptors on cells reduces the cellular uptake of Phl-erda@Ab.

**Figure 4:**
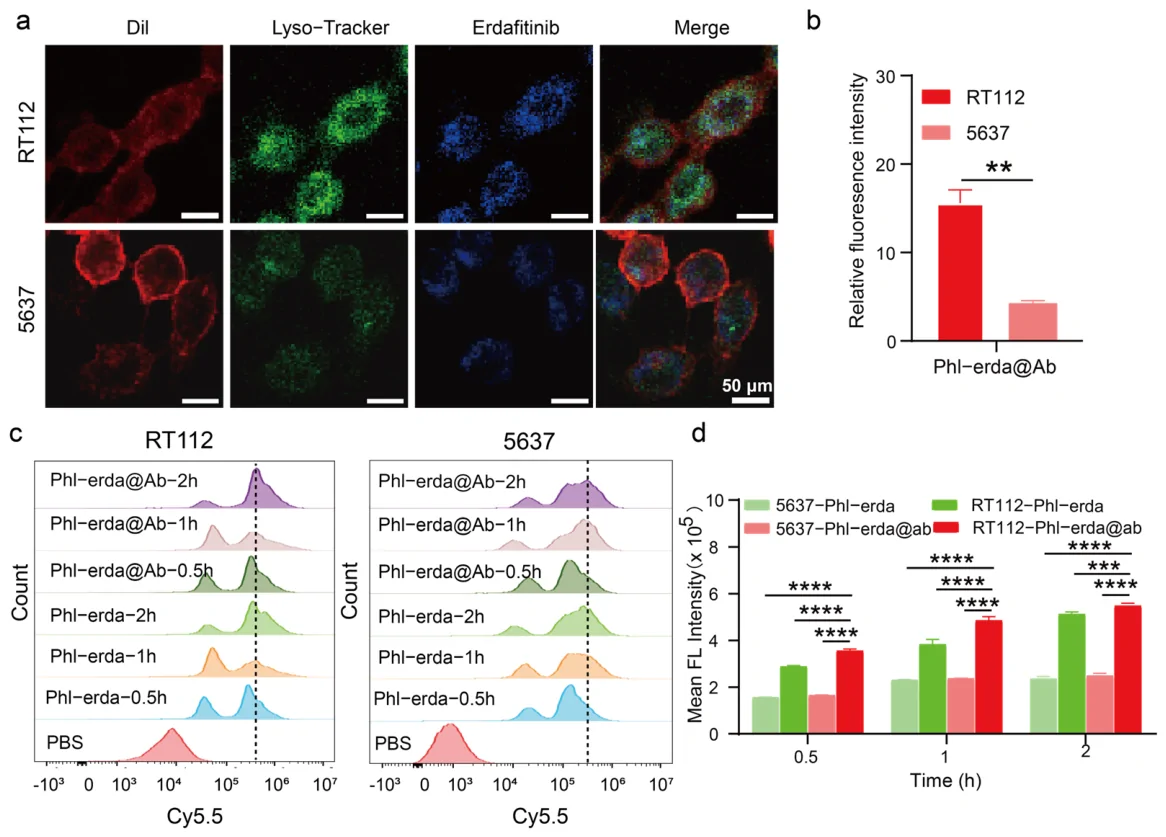
Cellular uptake of Phl-erda@Ab by RT112 cells. (**a**) CLSM images showing the cellular uptake of Phl-erda@Ab in RT112 and 5637 cells after incubation for 1 h. The cell membrane, lysosomes, and Phl-erda@Ab were labeled by Dil (red), Lyso-Tracker (green), and Cy5.5 (blue), respectively. Scale bar: 50 μm. (**b**) Semiquantification of fluorescence intensity of Phl-erda@Ab in cells. (**c**) Representative flow cytometry histograms and (**d**) quantitative analysis of Phl-erda@Ab fluorescence in cells. In Figure 4c, the dashed lines on the *y*-axis represent the peak fluorescence intensity positions of different groups at various time points, using Phl-erda@Ab-2h as the reference for comparison. This approach allows for a direct visual comparison of fluorescence intensity values under different treatment conditions. Data were analyzed using two-way ANOVA. Significance levels are indicated as follows: ** *p* < 0.01, *** *p* < 0.001, **** *p* < 0.0001.

To study the cellular uptake, both Phl-erda@Ab and Phl-erda were labeled with Cy5.5 (blue) and further incubated with RT112 cells, RT4 cells, and 5637 cells for different time intervals (0.5, 1, and 2 h). Subsequently, CLSM and flow cytometry were employed to analyze the changes in fluorescence intensity at different time intervals, respectively. As shown in the confocal images in Figure 4a and Figure S7a, Phl-erda@Ab exhibited significantly higher uptake in RT112 and RT4 cells than in 5637 cells. And Phl-erda@Ab (blue) and lysosomes (green) were clearly colocalized in RT112 and RT4 cells (Figure 4a and Figure S7a), with Pearson’s R values of 0.74 for RT112, 0.37 for RT4, and 0.22 for 5637 cells, indicating that Phl-erda@Ab is internalized into cells and accumulated in lysosomes. The mean fluorescence intensity in RT112 and RT4 cells was approximately 3 times higher than that in 5637 cells (Figure 4b and Figure S7b).

The flow cytometry results further demonstrated the preferential uptake. Flow cytometry histograms and MFI quantification results confirmed that the fluorescence intensity of Phl-erda@Ab in the RT112 cells was 1.7 times higher than that in 5637 cells. To further examine the intracellular uptake, we utilized the Cy5.5-labeled Phl-erda as control nanoparticles, which were incubated with RT112 cells. Flow cytometry revealed that the fluorescence intensity of Phl-erda@Ab in RT112 cells increased with incubation time. After 2 h of incubation, the fluorescence intensity of Phl-erda@Ab was ~1.3-fold greater than that of Phl-erda in RT112 (Figure 4c,d). These findings suggested that the coatings of anti-*FGFR3* antibodies on Phl-erda effectively facilitated the intracellular uptake of Phl-erda@Ab by *FGFR3*-overexpressing RT112 cells.

#### 2.2.3. Evaluation of the Antitumor Effect of Phl-erda@Ab on Rt112 Cells

**Figure 5:**
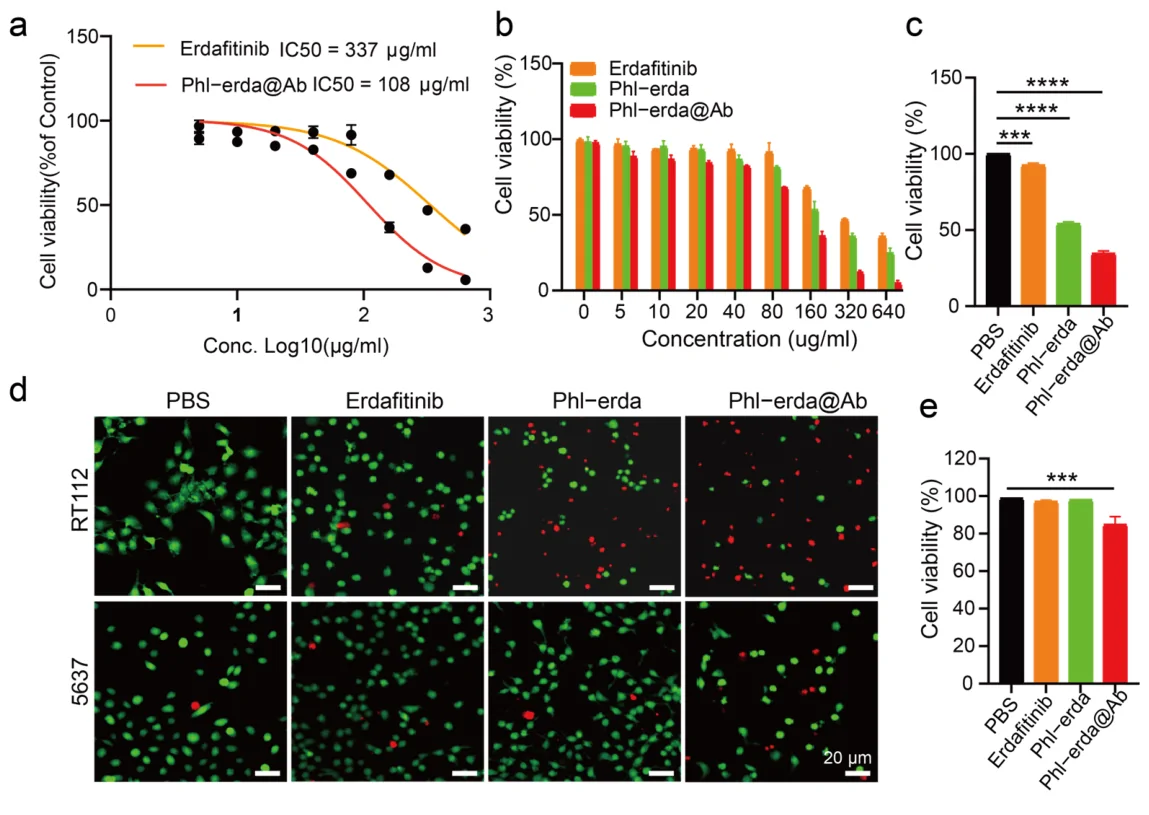
Evaluation of the antitumor effect of Phl-erda@Ab on RT112 cells in vitro. IC50 values of (**a**) erdafitinib and Phl-erda@Ab in RT112 cells. (**b**) Cytotoxicity of erdafitinib, Phl-erda, and Phl-erda@Ab against RT112 cells at various concentrations. (**c**) Relative survival rate of RT112 cells following treatment with the live-dead staining method. (**d**) CLSM images of RT112 and 5637 cells stained with calretinin AM (live cells, green) and PI (dead cells, red) after incubation with different treatment groups for 24 h. Scale bar: 20 μm. (**e**) Relative survival rate of 5637 cells following treatment with the live-dead staining method. Data were analyzed using one-way ANOVA. Significance levels are indicated as follows: *** *p* < 0.001, **** *p* < 0.0001.

To examine the antitumor activity of Phl-erda@Ab in vitro, we determined the IC50 values of Phl-erda@Ab and erdafitinib in RT112 cells. As shown in Figure 5a, the IC50 value of Phl-erda@Ab was 108 μg/mL, which is significantly lower than 337 μg/mL of erdafitinib. It is suggested that the Phl-erda@Ab located in lysosomes after cell uptake shows much more effective antitumor activity than erdafitinib in RT112 cells.

We performed cytotoxicity assays using a CCK-8 method to observe cell viability. As presented in Figures S2 and S8a, the viability of the RT112 and RT4 cells remained above 80% even after 24 h of incubation with high concentration (640 μg/mL) of Phl, suggesting the minimal cytotoxic effect of the Phl carriers. The cytotoxic effects of Erdafitinib, Phl-erda, and Phl-erda@Ab were further detected. As displayed in Figure 5b and Figure S8a, the cell viabilities of erdafitinib, Phl-erda, and Phl-erda@Ab- treatment groups decreased as the erdafitinib concentration increased from 0 to 640 μg/mL, showing a concentration-dependent trend. At the same high erdafitinib concentration of 640 μg/mL, the Phl-erda@Ab group showed the highest RT112 and RT4 cells survival inhibition rates of 94.82% and 97.42% in comparison with the erdafitinib (65.18% and 91.63%) and Phl-erda (75.44% and 93.46%) treated groups. It indicated that the Phl-erda@Ab had a significantly enhanced RT112 cell killing effect compared to erdafitinib and Phl-erda. In RT4 cells, compared with erdafitinib and Phl-erda, the inhibitory effect of Phl-erda@Ab on the cells is only slightly enhanced.

We further investigated the cytotoxic effect of Phl-erda@Ab using a live/dead cell staining assay. Notably, cell viability of RT112 and RT4 cells in the Phl-erda@Ab group was significantly reduced (approximately 34.7% and 56.04%) compared with the other groups, indicating superior antitumor efficacy (Figure 5c,d and Figure S8c,d). The viability of RT112 and RT4 cells treated with Phl-erda@Ab was 0.41-fold and 0.66-fold that of 5637 cells (Figure 5c,e and Figure S8d), suggesting that antibody modification of Phl-erda@Ab enhanced the inhibitory effect of erdafitinib on FGFR3-overexpressing RT112 and RT4 cells.

### 2.3. In Vivo Experiments

#### 2.3.1. *FGFR3*-Targeting Ability of Phl-erda@Ab in an Orthotopic Bca Mice

**Figure 6:**
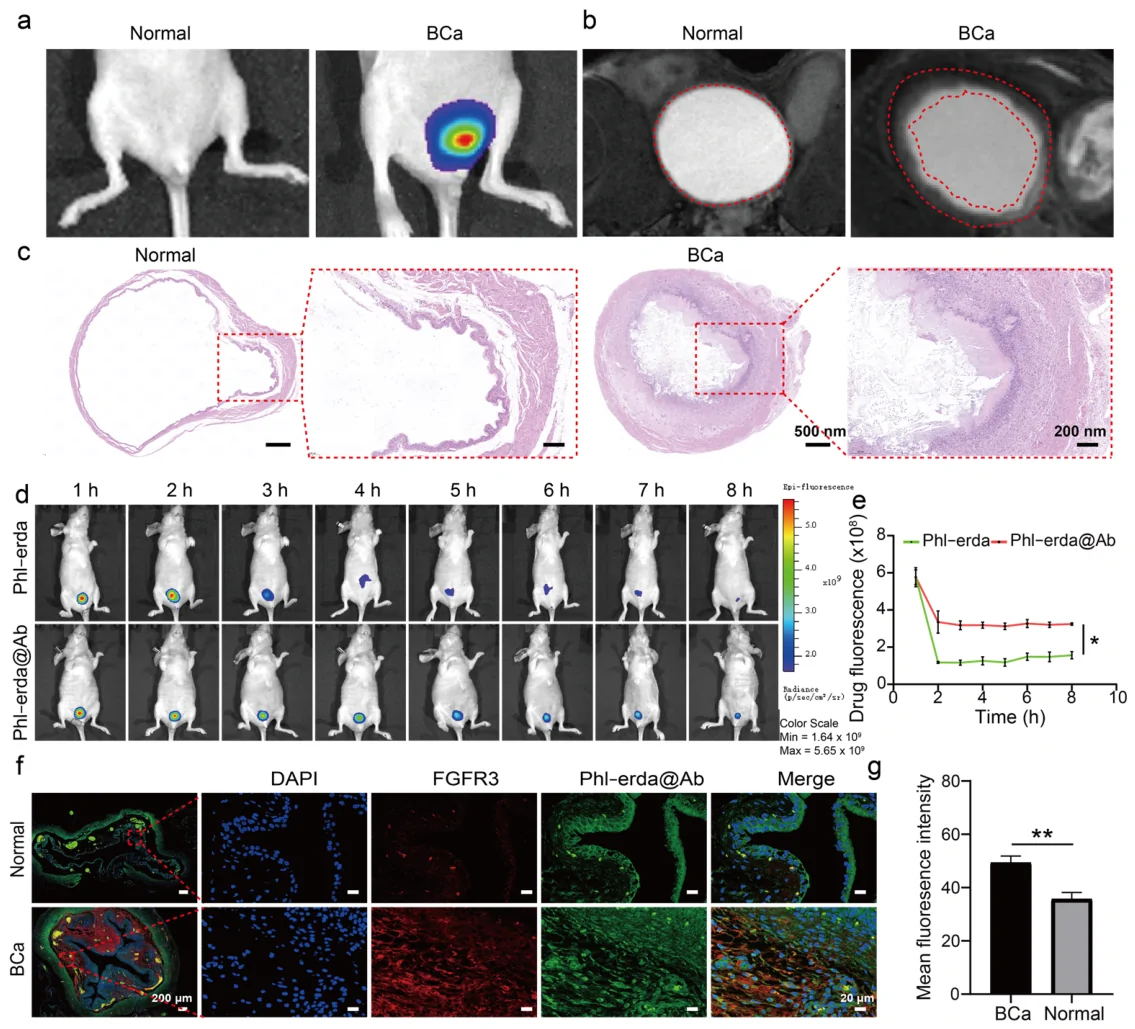
Orthotopic Bladder Cancer Model Establishment, Bladder Drug Retention, and In Vivo Targeting of Phl-erda@Ab to *FGFR3* in *BALB/c Nude Mice*. (**a**) Representative bioluminescence images of normal and bladder cancer-bearing *nude mice* at 14 days post-inoculation. (**b**) MRI images of normal and bladder cancer-bearing nude mice. The outermost red circle in the picture points to the bladder, and the inner red circle points to the tumour in the bladder. (**c**) H&E staining sections of bladder tissues from normal and tumor-bearing *nude mice*. Scale bars: 500 nm. (**d**) Fluorescence images and (**e**) quantitative analysis of fluorescence intensity in the bladder obtained at different time intervals following intravesical instillation of Cy5.5-labeled Phl-erda or Phl-erda@Ab, respectively. (**f**) Immunofluorescence staining images of *FGFR3* (red) and Phl-erda@Ab (green) in normal bladder tissue and BCa tissue of *mice*. Nuclei were stained with DAPI (blue). Scale bars: 20 μm. (**g**) Mean fluorescence quantification analysis of Phl-erda@Ab. An unpaired *t*-test was performed to analyze the significant difference between the Normal and BCa groups (*n* = 3). Data were analyzed using a two-tailed Student’s *t*-test. and 200 nm. Data were analyzed using a two-tailed Student’s *t*-test (*n* = 3). Significance levels are indicated as follows: * *p* < 0.05, ** *p* < 0.01.

To investigate the in vivo therapeutic effect of Phl-erda@Ab, we established an orthotopic BCa model by intra-bladder perfusion of RT112-Luc cells in *nude mice*. Bioluminescence imaging was employed to observe the tumor growth weekly. Figure S6a revealed a clear Luc bioluminescence signal in the bladder, which confirmed the successful establishment of orthotopic BCa mice after intravesical perfusion on day 14 (Figure 6a). The ultra-high field (9.4 Tesla) Magnetic resonance imaging (MRI) T2-weighted images showed the annular low-signal tumor lesions around the bladder wall of *mice* (Figure 6b). And H&E staining images revealed extensive infiltration of atypical tumor cells in the mucosal and muscular layers of the bladder in *mice* (Figure 6c). These results collectively confirmed that the approach for establishing orthotopic BCa mice was successful and reliable, which is very beneficial for subsequent evaluation of the therapeutic efficacy of Phl-erda@Ab.

Before evaluating the in vivo therapeutic efficacy, we investigated the retention time of Phl-erda@Ab after intravesical injection into the bladder of mice. The Cy5.5-labeled Phl-erda and Phl-erda@Ab (4.81 mg/kg) were perfused into the bladders of BCa *mice*, and bladder fluorescence signals were dynamically monitored over 8 h after perfusion by fluorescence imaging. The fluorescence intensity of Phl-erda@Ab in the bladder at 2 h post-perfusion remained above 60%, which was significantly higher than that (20%) in the Phl-erda treated group (Figure 6d,e). It indicated that the anti-*FGFR3* antibodies on Phl-erda@Ab effectively prolonged the liposome retention time in the bladder.

We further evaluated the in vivo tumor-targeting ability of Phl-erda@Ab that was labeled with FITC (green). After intravesical instillation of Phl-erda@Ab, we conducted immunofluorescence staining of bladder tissue sections of *mice.* As shown in Figure 6f, the red fluorescence intensity of *FGFR3* in BCa mice was significantly higher compared with the normal bladder tissue in healthy *mice*, indicating that *FGFR3* expression was upregulated in tumor tissue in BCa *mice*. The green fluorescence signal of Phl-erda@Ab and the red fluorescence signal of *FGFR3* protein were colocalized in BCa tissue. In contrast, the localization of green and red fluorescence cannot be observed in normal bladder tissue. As shown in Figure 6f,g, the content of Phl-erda@Ab in tumor-bearing bladder tissues was higher than that in healthy bladder tissues. These results suggested that the Phl-erda@Ab showed enhanced accumulation at the RT112 tumor sites in BCa-bearing *mice* with *FGFR3*-overexpressing tumors.

#### 2.3.2. Phl-erda@Ab-Triggered BCa Therapeutic Efficacy

**Figure 7:**
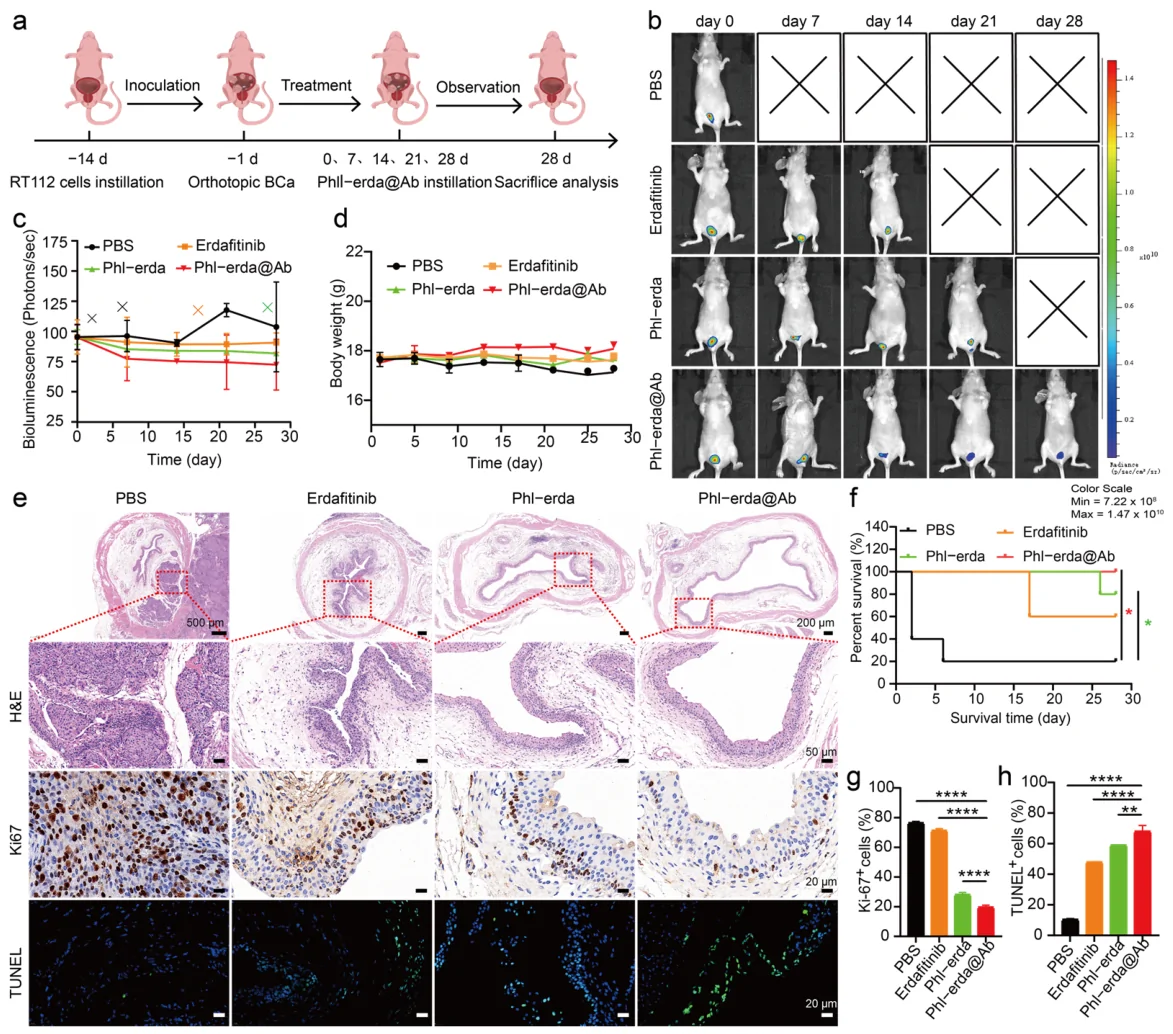
Intravesical instillation of Phl-erda@Ab showed a significant therapeutic effect on orthotopic BCa models. (**a**) Schematic diagram of tumor treatment in vivo. (**b**) Quantified diagram of the bladder in different treatment groups (PBS, erdafitinib, Phl-erda, and Phl-erda@Ab) at day 0, 7, 14, 21, and 28. (**c**) Quantified bioluminescence signals of individual mice over time; “×” denotes animals that died during the observation period (black, PBS; orange, erdafitinib; green, Phl-erda). Data are presented descriptively without inferential statistics (see Discussion). (**d**) Body weight changes in mice in each group during the treatment period (*n* = 3). (**e**) Representative images of H&E staining, Ki-67 immunohistochemistry, and TUNEL immunofluorescence of bladder tumors from *mice* after different treatments. (**f**) Survival curves were analyzed using the Kaplan–Meier method with the log-rank (Mantel–Cox) test for comparisons between PBS vs. Phl-erda and PBS vs. Phl-erda@Ab. Quantitative analysis of (**g**) Ki-67-positive cells and (**h**) TUNEL-positive cells. One-way ANOVA with Tukey’s post-hoc test was used to compare the Phl-erda@Ab group against PBS, free erdafitinib, and non-targeted Phl-erda groups, respectively. (*n* = 5). Data were analyzed using one-way ANOVA. Significance levels are indicated as follows: * *p* < 0.05, ** *p* < 0.01, **** *p* < 0.0001.

We further performed the assessment of in vivo therapeutic efficacy. Tumor-bearing *nude mice* were randomly divided into 4 groups, including the PBS, erdafitinib, Phl-erda, and Phl-erda@Ab treatment groups (Figure 7a). The drug dosage of erdafitinib for intravesical instillation was 4.81 mg/kg in erdafitinib, Phl-erda, and Phl-erda@Ab groups. The tumor growth was monitored by bioluminescence imaging weekly (Figure 7b). The results exhibited that the erdafitinib treatment showed limited antitumor effects, confirming that erdafitinib-based therapy is insufficient for inhibiting tumor growth (Figure 7b,c). Animals in the Erdafitinib group began to die on day 17 of treatment, with the survival rate dropping to 60% and remaining at that level between days 17 and 28 (Figure 7f). In terms of therapeutic effect, this group exhibited almost no significant tumour inhibition throughout the entire observation period (Figure 7c). In contrast, animal deaths in the PBS group began as early as day 2, with the survival rate decreasing to 20% by day 6, and the final overall survival rate was also 20% (Figure 7f). No therapeutic effect was observed, and tumour volume showed an increasing trend (Figure 7c). Phl-erda caused a modest cancer cell killing effect. Animals in the Phl-erda group began to die on day 26, with the survival rate dropping to 80%, and it remained at this level until the end of the treatment, but the overall efficacy throughout the treatment period was very limited. In contrast, no deaths occurred in the Phl-erda@Ab group during the treatment period, with a survival rate of 100%; compared with the PBS group, this group exhibited a certain tumour growth inhibition effect throughout the treatment cycle, but in the Phl-erda@Ab group itself, the efficacy from days 7 to 21 showed almost no significant improvement. The Phl-erda@Ab treatment resulted in tumor growth arrest, demonstrating the significant therapeutic efficacy of combining specific tumor-targeting with effective cell uptake of erdafitinib for superior intravesical therapy. Notably, after 28 days of treatment, the fluorescence intensity in the Phl-erda@Ab group was approximately 1-fold lower than that in the Phl-erda group, further demonstrating the enhanced antitumor activity of Phl-erda@Ab treatment. Body weight was monitored every four days to assess systemic toxicity (Figure 7d). Throughout the entire course of treatment, the Phl-erda@Ab group showed slightly increased body weight. In contrast, the Phl-erda and erdafitinib group exhibited stable body weight during the treatment period and the weight of the animals in the PBS group slightly dropped. This indicates that under local perfusion pathways, the effect of the drug on the overall body weight of animals is generally minimal. The tumor weights suggested that the Phl-erda@Ab treatment resulted in the most efficient tumor suppression or tumor eradication compared to other groups. And the Phl-erda@Ab treatment due to its improved antitumor effect significantly prolonged the survival of BCa *mice* (Figure 7f).

After treatment, H&E staining, TUNEL apoptosis detection, and Ki67 immunohistochemical staining of bladder sections from each group were performed to further investigate therapeutic effects (Figure 7e). The H&E staining results of bladder sections confirmed that the Phl-erda@Ab treatment induced the most superior tumor inhibition effect. The Ki67 staining images demonstrated that the Phl-erda@Ab group exhibited the least Ki67-positive cells in the tumor, suggesting the tumor proliferation inhibition (Figure 7e,g). In addition, the Phl-erda@Ab group showed the most pronounced cell apoptosis, as displayed in the TUNEL-staining results (Figure 7e,h).

Further, H&E staining of the major organs, including the heart, liver, spleen, lung, and kidneys of mice from different groups was performed to evaluate the systemic biosafety. As shown in Figure S3, no obvious tissue damage or pathological alterations in the Phl-erda@Ab group were observed in any of the major organs in comparison with the other groups, indicating that the Phl-erda@Ab-based intravesical instillation therapy had an acceptable safety profile in *mice*.

## 3. Discussion

BCa imposes a substantial global disease burden, with radical cystectomy remaining the cornerstone for MIBC yet irrevocably affecting urinary function and quality of life [32,33]. Although trimodal bladder-preserving therapy offers an organ-sparing alternative, its applicability is restricted to highly selected patients with favorable baseline characteristics, underscoring the urgent need for effective, broadly accessible locoregional strategies [34,35,36]. In the precision oncology era, *FGFR3* has emerged as a validated oncogenic driver across the entire BCa spectrum, from non-muscle-invasive to muscle-invasive subtypes. Erdafitinib, which received FDA approval as the pioneering *FGFR3*-targeted agent for managing cisplatin-refractory MIBC, has exhibited sustained objective responses in clinical settings [36]. Yet when given orally or intravenously, this agent often triggers dose-limiting toxicities, such as hyperphosphatemia and oral mucositis, that restrict how much drug can be safely delivered and make long-term adherence difficult [26,37,38].

The bladder’s anatomy makes intravesical instillation an obvious way around this efficacy-versus-toxicity problem. Delivering the drug directly to the mucosal surface achieves high local concentrations while keeping systemic exposure low, which effectively separates antitumor effects from unwanted side effects elsewhere [39,40]. This approach sounds promising in theory, yet current preclinical models still fall short of capturing the bladder’s unique architecture, physiology, and microenvironment. Consequently, how well intravesical nanocarrier strategies might translate to the clinic remains largely unclear.

To fill this gap, we designed Phl-erda@Ab, a liposomal system that combines pH-triggered release, *FGFR3*-targeting capability, and erdafitinib loading in one construct. These three features work together, making the platform well-suited for intravesical bladder cancer therapy. First, we designed the pH-sensitive imide bonds to confer acid-triggered erdafitinib release by degrading liposomes [28,41,42]. The rationale for exploiting acidic conditions as a release trigger is rooted in the distinctive pathophysiology of BCa. Unlike normal urothelial cells that reside in a relatively neutral luminal environment (pH 6.5–7.0), malignant bladder tissues often exhibit an acidic extracellular milieu due to upregulated glycolysis, heightened lactate production, and impaired perfusion-collectively known as the Warburg effect-which drives local pH values as low as 6.0–6.5 [43,44]. It facilitated efficient drug release within acid lysosome for maximizing tumor cell killing effect while minimizing the nonspecific release and cytotoxicity of erdafitinib to normal cells within the neutral bladder lumen.

Beyond the extracellular acidic niche, a steeper pH gradient exists along the intracellular endolysosomal route. Following receptor-mediated endocytosis, the nanocarrier–*FGFR3* complex is trafficked through early endosomes (pH-6.0) to late endosomes (pH-5.0) and finally lysosomes (pH 4.5–5.0) [45,46]. This gradient offers a dual-layer trigger: the weakly acidic tumor interstitium initiates surface drug release, while the strongly acidic lysosome ensures rapid liberation of erdafitinib at its cytoplasmic target (FGFR3), avoiding premature sequestration or degradation. By incorporating acid-labile imide bonds, Phl-erda@Ab achieves “dual-pH responsiveness”, involving the release of a moderate payload in the peritumoral niche to prime tumor cells, and the majority inside lysosomes upon internalization. The coating of anti-*FGFR3* antibodies endows Phl-erda@Ab with active recognition and binding of the *FGFR3* overexpressed on tumor cells [47,48,49,50].

Further, the transmembrane *FGFR3* protein and mucosal adhesion liposome synergistically enhanced the endocytic uptake and lysosome colocalization of erdafitinib, facilitating direct cytoplasmic drug delivery and prolonging the tumor site accumulation efficacy and retention time. We referenced previous studies by employing pH-sensitive liposomes to encapsulate the drug and conjugating *FGFR3* antibodies onto their surface [51]. This design, combined with the intravesical route of administration, offers several critical advantages for bladder cancer therapy. First, the pH-sensitive liposomes enable targeted delivery to *FGFR3*-overexpressing tumor cells and facilitate pH-triggered drug release within the acidic lysosomal environment, which increases the local drug concentration at the tumor site and thereby enhances the inhibitory and cytotoxic effects against tumor cells. Second, intravesical instillation allows direct delivery of the therapeutic agent to the bladder tumor, achieving high local drug concentrations while minimizing systemic exposure and associated side effects. This localized approach also circumvents first-pass metabolism, improves bioavailability, and enables repeated dosing with reduced systemic toxicity.

Building on this rational design, we further systematically evaluated the antitumor efficacy of Phl-erda@Ab in both in vitro and in vivo models. The uptake of Phl-erda@Ab by *FGFR3*-overexpressing RT112 cells was markedly higher than that by 5637 cells with low *FGFR3* expression, confirming receptor-mediated internalization. The IC50 of Phl-erda@Ab in RT112 cells (108 μg/mL) was significantly lower than that of free erdafitinib (337 μg/mL), demonstrating that targeted intracellular delivery amplified cytotoxic potency specifically in *FGFR3*-overexpressing BCa cells.

The efficacy of Phl-erda@Ab probably lies in two aspects: (i) enhanced cytotoxicity in sensitive cells and (ii) sustained liposome retention in the bladder. In RT112 cells, for instance, the apoptotic index peaked with this formulation, whereas the same treatment elicited only a muted response in 5637 cells, confirming that target engagement is key. In the orthotopic model, the retention data were equally compelling, with more than 60% of the instilled dose remaining in the bladder at 2 h, far outperforming the non-targeted counterpart. This improved staying power translated into clear therapeutic benefits. By day 28, bioluminescence in the Phl-erda@Ab group had dropped substantially compared with Phl-erda, while free erdafitinib barely suppressed tumor growth and looked no different from the untreated controls. Histopathological analysis also demonstrated maximal tumor necrosis, the highest TUNEL-positive apoptotic burden, and the lowest Ki67 proliferation index in the Phl-erda@Ab group. Body weight remained stable, and no significant pathological damage to major organs was observed in the Phl-erda@Ab group, underscoring an acceptable safety profile at the administered dose.

Collectively, these findings establish Phl-erda@Ab as a precision intravesical nanoplatform that resolves the central paradox of *FGFR3*-targeted therapy, namely, how to achieve potent bladder tumor inhibition without incurring systemic toxicity. The efficient treatment strategy of BCa by intravesical installation of Phl-erda@Ab, coupled with the absence of systemic adverse effects, may expand the therapeutic window of this approved agent beyond its current indication.

In addition, several caveats warrant acknowledgment and shape our future directions. First, while the therapeutic efficacy of Phl-erda@Ab has been established, the underlying mechanistic landscape remains incompletely characterized. We acknowledge that, while the antiproliferative effects observed are consistent with *FGFR3*-targeted delivery, the direct inhibition of *FGFR3* downstream signaling pathways (e.g., p-ERK1/2 and p-AKT) was not experimentally validated in the RT112 model used in the present study. We therefore refrain from attributing the observed biological effects solely to *FGFR3* signaling blockade. This important mechanistic aspect represents a key direction for our ongoing and future investigations. Meng et al. demonstrated that erdafitinib significantly suppressed p-ERK1/2 and p-AKT in *FGFR3*-mutant BCa cells (SW780 and UMUC-14), thereby inhibiting proliferation, migration, and invasion [52], lending mechanistic plausibility to similar pathway involvement in our study. Future work should leverage single-cell transcriptomics and spatially resolved proteomics to dissect the precise intracellular trafficking of the nanosystem, particularly the kinetics of lysosomal escape, cytoplasmic drug liberation, and *FGFR3* downstream signaling blockade (e.g., MAPK/PI3K/STAT axis), as well as to elucidate the tumor microenvironmental remodeling. Second, the platform’s generalizability across the *FGFR3*-driven oncologic spectrum remains unvalidated: additional *FGFR3*-high bladder cancer lines (e.g., J82 and UM-UC-3) and other *FGFR3*-mutant malignancies must be examined. Additional FGFR3-high bladder cancer lines such as J82 and UM-UC-3, along with other *FGFR3*-mutant malignancies, need to be examined to get a clearer sense of where this approach might fit. Even though our formulation exhibits favorable stability in artificial urine over the tested timeframe, further validation in real urine samples or under dynamic in vivo conditions would be valuable for clinical translation. The *mouse* bladder also differs substantially from human anatomy in size, urine composition, and mucosal thickness. Larger animal models like porcine or canine would be better suited to refining instillation protocols and checking for systemic drug leakage before moving forward. We acknowledge that the sample size of our animal data is relatively small, and future studies will employ larger sample sizes (e.g., n ≥ 6 per group) to strengthen the statistical confidence and reproducibility of the therapeutic outcomes. It should be noted that, owing to the small group size and informative dropout caused by early animal deaths, the longitudinal bioluminescence data alone were insufficient to statistically differentiate the Phl-erda@Ab group from the other groups. The therapeutic efficacy of Phl-erda@Ab is therefore supported by concordant and statistically significant differences in overall survival, the Ki67 proliferation index, and the TUNEL-positive apoptotic index, which together provide independent and mutually reinforcing evidence for the antitumor effect of Phl-erda@Ab. In the receptor blocking experiments, uptake of Phl-erda@Ab was significantly reduced upon *FGFR3* blockade not only in RT112 and RT4 cells with high *FGFR3* expression but also, to a lesser extent, in 5637 cells with relatively low *FGFR3* expression. We attribute the blocking effect in 5637 cells to two non-exclusive factors. First, 5637 cells express *FGFR3* at low but detectable levels rather than being completely negative; at low receptor density, partial blockade of this residual receptor pool is sufficient to produce a measurable reduction in uptake, which reflects attenuated receptor-mediated internalization rather than a loss of targeting specificity. Second, a fraction of liposome uptake in 5637 cells is likely mediated by non-specific pathways, such as adsorptive endocytosis of the liposomal surface and, under serum-containing conditions, protein corona–mediated transport, which operates independently of *FGFR3* and therefore persists after receptor blockade [53,54,55]. Accordingly, the ‘residual uptake’ observed in blocked 5637 cells most plausibly represents this non-specific background. Finally, the long-term biocompatibility and nanotoxicological profile of repeated intravesical Phl-erda@Ab administration remain undefined. The current safety evaluation mainly relies on histopathological examination, and pharmacokinetic data following intravesical administration have not yet been provided. In future studies, we will determine erdafitinib concentrations in plasma and major tissues (e.g., heart, liver, spleen, lung, and kidney) at different time points after intravesical administration of Phl-erda@Ab, in order to obtain direct evidence of systemic exposure and further improve the safety evaluation system. Specifically, prospective assessment must evaluate chronic nanomaterial deposition in the lamina propria, antibody-mediated immunogenicity, off-target binding to normal urothelium, and the potential for lysosomal dysfunction or inflammatory fibrosis following multi-cycle instillations. These questions are essential to address for any IND-enabling package.

## 4. Materials and Methods

### 4.1. Material Preparation and Characterisation

#### 4.1.1. Materials and Reagents

All reagents used in this work were of commercial origin. Erdafitinib (purity 99.37%) was obtained from Shandong Sparkjade (Jinan, China), while lecithin came from Shanghai Yuanye Bio-Technology Co., Ltd. (Shanghai, China). For liposome preparation, we sourced DSPE-Hyd-PEG2000, the antibody conjugation kit, and Cy5.5 fluorescent dye from Xi’an Ruixi Biological Technology Co., Ltd. (Xi’an, China). The anti-*FGFR3* antibody for surface modification was purchased from Proteintech Group, Inc. (Wuhan, China). For the evaluation of cytotoxicity and apoptosis, the CCK-8 assay kit and the Annexin V-FITC/PI Apoptosis Detection Kit were sourced from Dojindo Laboratories (Kumamoto, Japan) and Beijing BailaiBo/Beijing BioLaiBo Technology Co., Ltd. (Beijing, China), respectively. Trypsin-EDTA and FITC were obtained from Beijing Solarbio Science & Technology Co., Ltd. (Beijing, China). Cell culture was performed using RPMI 1640 medium (Gibco, Thermo Fisher Scientific (Waltham, MA, USA)) supplemented with fetal bovine serum (Sangon Biotech (Shanghai) Co., Ltd. (Shanghai, China)). The fluorescent probes DiI and Lyso-Tracker Green were procured from Beyotime Biotechnology (Shanghai, China).

#### 4.1.2. Preparation of Phl-Erda@ab

The pH-responsive, erdafitinib-loaded, anti-*FGFR3* antibody-conjugated liposomes (Phl-erda@Ab) were fabricated via the thin-film hydration method [56,57]. Briefly, lecithin (10 mg), DSPE-Hyd-PEG_2000_ (10 mg), erdafitinib (10 mg), and Cy5.5 (40 μL of a 500 μg/mL stock solution) were co-dissolved in 4 mL of chloroform in a round-bottom flask. We began preparing Phl-erda by evaporating the organic solvent under reduced pressure at 35 °C using a rotary evaporator. This gave a thin, uniform lipid film on the flask wall. The film was then hydrated with 4 mL ultrapure water, followed by another 20 min of rotary evaporation, yielding an erdafitinib-loaded liposomal suspension that responds to pH changes. This pH sensitivity comes from the hydrazone bond (–C=N–NH–) in DSPE-Hyd-PEG2000, which is acid-labile: it breaks under acidic conditions and destabilizes the liposomal membrane accordingly.

To confer active tumor-targeting capability, we covalently linked FGFR3-specific antibodies onto the liposome surface using a commercially available phospholipid-based conjugation kit, following the manufacturer’s protocol. In brief, 1 mL of the pre-formed Phl-erda suspension was transferred to a sterile 1.5 mL tube, then mixed with 10 μL activation buffer and 20 μg anti-*FGFR3* antibody. This mixture was gently rotated at 4 °C for 2 h in the dark to allow selective covalent attachment. The resulting targeted, pH-responsive formulation was designated Phl-erda@Ab. As a control, we also prepared blank liposomes (Phl) using the same thin-film hydration approach but without adding erdafitinib. These antibody-free, drug-free carriers were used as negative controls to assess any vehicle-related effects.

#### 4.1.3. Determination of Antibody Conjugation Efficiency by BCA Assay

The antibody conjugation efficiency of Phl-erda@Ab was determined using a bicinchoninic acid (BCA) protein assay. Unconjugated antibodies were removed from the Phl-erda@Ab preparation by dialysis before protein quantification. After purification, the liposomes were disrupted with 1% Triton X-100 (30 min, room temperature) to release the conjugated protein. The protein concentration in the supernatant was measured using the BCA assay according to the manufacturer’s instructions. Each sample (20 µL) was combined with BCA working reagent (200 µL) in a 96-well format. The plate was incubated at 37 °C for 30 min, and the absorbance was recorded at 570 nm using a microplate reader. The antibody concentration was calculated from the standard curve. Since the total amount of antibody initially added was known, the conjugation efficiency was calculated using the following formula [58,59]:

(1) $$A_{coupling\;antibody}=A_{totally\;added\;antibody}-A_{antibody\;purified\;liposomes}$$

(2) $$E_{coupling}=\frac{A_{antibody\;bound\;to\;liposomes}}{A_{total\;added\;antibody}}\times 100\%$$

where $A_{coupling\;antibody}$, $A_{totally\;added\;antibody}$, $A_{antibody\;purified\;liposomes}$, and $A_{antibody\;bound\;to\;liposoes}$ represent the amount of coupling antibody, total added antibody, antibody purified liposomes, and antibody bound to liposomes, respectively. $E_{coupling}$ denotes the coupling efficiency.

#### 4.1.4. Characterization

Two complementary analytical approaches were applied to characterize the prepared formulations. For morphological assessment, transmission electron microscopy (TEM; Talos F200S, Thermo Fisher Scientific (Waltham, MA, USA))was utilized, with samples placed on carbon-coated copper grids and negatively contrasted using 2% phosphotungstic acid. For physicochemical parameters, dynamic light scattering (DLS; NanoBrook Omni, Brookhaven Instruments Corporation (Holtsville, NY, USA)) provided measurements of hydrodynamic diameter, polydispersity index, and zeta potential, all recorded at 25 °C from three replicate runs. The drug loading and encapsulation efficiency of Phl-erda@Ab were determined by LC-MS/MS (SCIEX Triple Quad™ 6500+ System, SCIEX (Framingham, MA, USA)) as specified in Section 4.1.5.

Phl, Phl-erda, and Phl-erda@Ab were synthesized according to the method described in Section 4.1.2 and stored in artificial urine for zeta potential and DLS analysis. In addition, the stability of Phl-erda was evaluated by DLS on days 1, 4, and 8 after synthesis. All data were recorded at 25 °C in triplicate.

#### 4.1.5. Determination of Drug Loading and Encapsulation Efficiency

To determine the drug loading efficiency, Phl-erda (encapsulated erdafitinib) was removed by dialysis against phosphate-buffered saline (PBS, pH 7.4) for 6 h at 4 °C using a dialysis membrane with a molecular weight cutoff (MWCO) of 2000 Da (Xi’an Haoyang Biotechnology Co., Ltd., Xi’an, China). The dialyzed Phl-erda suspension was then lysed by the addition of a 10-fold volume of methanol, followed by sonication for 30 min to disrupt the lipid bilayer and completely release the encapsulated drug. The erdafitinib concentration in the resulting solution was quantified by LC-MS/MS. Drug loading content (DLC) and the cumulative drug release at time t (denoted as $Q_{t}$) were calculated according to the following equations:

(3) $$\mathrm{DLC}\;(\%)\;=\;\frac{m_{erdafitinib}}{m_{totallipids}+m_{erdafitinib}}\times 100\%$$

(4) $$Q_{t}(\%)=\frac{C_{t}.v_{s}+\sum_{i=1}^{t-1}C_{t}.v_{s}}{m_{totalerdafitinibfed}}\times 100\%$$

where $m_{erdafitinib}$ represents the mass of erdafitinib successfully loaded into the liposomes, $m_{totallipids}$ represents the total mass of phospholipids (lecithin and DSPE-Hyd-PEG_2000_), where $Q_{t}$ represents the cumulative drug release rate at time t, $C_{t}$ represents the Erdafitinib concentration in the release medium at time t, $V_{s}$ represents the volume of each withdrawn sample and $\sum_{i=1}^{t-1}C_{t}.v_{s}$ represents the total mass of Erdafitinib removed in all previous sampling events and $m_{totalerdafitinibfed}$ represents the initial mass of erdafitinib added during preparation.

#### 4.1.6. In Vitro pH-Triggered Drug Release Kinetics

The pH-responsive drug release profiles of Phl-erda were evaluated using the dialysis bag diffusion method across three biologically relevant pH conditions: pH 7.4 (physiological blood/healthy tissue), pH 6.5 (mildly acidic bladder urine), and pH 5.3 (lysosomal compartments).

Erdafitinib stock solutions (1000 μg/mL) were prepared in PBS at pH 5.3, 6.5, and 7.4, respectively, and serially diluted to generate gradient standard solutions ranging from 0.1 to 50.0 μg/mL. The chromatographic peak areas were measured by LC-MS/MS, and linear regression equations were established for each pH condition to ensure accurate quantification across different release media.

We placed 1 mL of Phl-erda suspension inside a dialysis bag (MWCO 2000 Da) and submerged it in 30 mL release medium—PBS containing 1% methanol (*v*/*v*)—adjusted to pH 7.4, 6.5, or 5.3. The setup was incubated on an orbital shaker at 100 rpm and 37 ± 0.5 °C. At set time points (0, 1, 2, 4, 6, 8, 12, and 24 h), we removed 1 mL from the external buffer and immediately replaced it with an equal volume of fresh, pre-warmed medium to maintain sink conditions. Erdafitinib concentrations in the collected samples were then determined by LC-MS/MS, and cumulative release percentages were calculated relative to the total drug loaded.

### 4.2. In Vitro Biological Evaluations

#### 4.2.1. Cell Lines and Culture Conditions

The *FGFR3*-overexpressing RT112 and RT4 BCa cell lines were used for in vitro experiments, while the 5637 cell line without expression of *FGFR3* was used as a control. RT112 and RT4 cells were obtained from Shanghai Yuchi Biotechnology Co., Ltd. (Shanghai, China). 5637 and the luciferase-tagged RT112-Luc variant came from the National Collection of Authenticated Cell Cultures (Shanghai, China). RT4 cells were maintained in McCoy’ s 5A supplemented with 20% FBS and 1% antibiotics, incubated at 37 °C under 5% CO_2_ in a humidified atmosphere. RT112 and 5637 cells were maintained in RPMI 1640 supplemented with 10% FBS and 1% antibiotics, incubated at 37 °C under 5% CO_2_ in a humidified atmosphere. The medium was changed every two days. For passaging, cells were harvested at 80–90% confluence by 0.25% trypsin-EDTA treatment, then spun down at 1000 rpm for 5 min and replated at a 1:3 ratio.

#### 4.2.2. Quantitative Real-Time PCR (qPCR) Analysis of *FGFR3* Expression

We compared *FGFR3* expression in RT112 and 5637 cells by qPCR. Total RNA was extracted with TRIzol reagent (Invitrogen, Thermo Fisher Scientific (Waltham, MA, USA)) following the supplier’s protocol, and sample purity was checked via A260/A280 absorbance ratios on a NanoDrop One (Thermo Fisher Scientific (Waltham, MA, USA)). For reverse transcription, 1000 ng RNA was converted to cDNA using the HiFiScript kit (CWBIO) at 42 °C for 15 min, then the reaction was stopped at 85 °C for 5 min. qPCR was carried out with SYBR Green Master Mix (Y&K Bio) on a real-time thermocycler: an initial 10 s denaturation at 94 °C, followed by 40 cycles of 94 °C for 10 s and 72 °C for 20 s [56,57]. Relative FGFR3 mRNA levels were calculated against GAPDH using the 2^(–ΔΔCt) method.

#### 4.2.3. Western Blot Analysis of *FGFR3* Protein Expression

*FGFR3* protein expression was detected by Western blotting. RT112, RT4, and 5637 cells in the logarithmic growth phase were collected. Total protein was extracted using RIPA lysis buffer, and protein concentrations were determined. Equal amounts of protein samples were separated by SDS-PAGE and then transferred onto PVDF membranes. The membranes were blocked with 5% non-fat milk at room temperature for 1 h. They were then incubated with a primary antibody against *FGFR3*, followed by an HRP-conjugated secondary antibody. Protein bands were visualized using an ECL chemiluminescent substrate. GAPDH was used as an internal control. Relative *FGFR3* protein expression levels among the three cell lines were compared by densitometric analysis.

#### 4.2.4. Evaluation of *Fgfr3*-Targeting of Phl-Erda@ab

Phl-erda@Ab was functionalized with FITC-conjugated anti-*FGFR3* antibody to enable fluorescent tracking of its cellular binding. RT112 cells were exposed to the FITC-labeled formulation for 1 h, followed by DAPI counterstaining. Confocal fluorescence microscopy (Zeiss LSM 980, Carl Zeiss AG (Oberkochen, Germany)) was then performed to assess the distribution and intensity of the fluorescent signals, thereby indicating the extent of active targeting. Integrated fluorescence intensity was measured within manually delineated regions of interest (ROIs) using ImageJ/Fiji software (version 2.16.0/1.54p), and background fluorescence was subtracted according to the corrected total cell fluorescence (CTCF) formula:

Corrected total cell fluorescence (CTCF) = integrated density − (area of selected ROI × mean background fluorescence).

The CTCF of the target signal (FGFR3) was then normalized to the CTCF of DAPI nuclear staining. This normalization yielded the relative fluorescence intensity (RFI) for each cell. Each experiment was repeated at least three times. Data are presented as mean ± SEM. Statistical comparisons between two groups were performed using an unpaired two-tailed Student’s *t*-test, and a *p*-value < 0.05 was considered statistically significant.

#### 4.2.5. Evaluation of Cell Uptake of Phl-erda@Ab

To visualize nanoparticle intracellular distribution, RT112, RT4 and 5637 cells plated in confocal dishes (1.5 × 10^5^ cells/dish, overnight) were treated with Cy5.5-labeled Phl-erda@Ab (100 μg/mL) for 1 h at 37 °C in the dark. Following PBS washing, cells were sequentially stained with LysoTracker Green (1:15,000, 1 h) to mark acidic vesicles and DiI (5 μM, 20 min) to delineate the plasma membrane. Confocal microscopy images were then acquired to reveal the relative positions of Cy5.5-labeled nanoparticles (blue), endo/lysosomes (green), and membranes (magenta). Regions of interest (ROIs) were manually delineated based on the Dil-labeled cell membrane contours using ImageJ/Fiji software. The integrated fluorescence intensity of erdafitinib was measured within each ROI, and background fluorescence was measured in a cell-free area adjacent to each ROI. The corrected total cell fluorescence (CTCF) was calculated using the following formula:

CTCF = integrated density − (ROI area × mean background fluorescence).

The relative fluorescence intensity per cell was expressed as the CTCF value, and the average value for each group was derived from all measured cells. Each experiment was repeated at least three times. Data are presented as mean ± SEM. Statistical comparisons between two groups were performed using an unpaired two-tailed Student’s *t*-test, and a *p*-value < 0.05 was considered statistically significant.

For uptake kinetics analysis, the three cell lines were incubated for 0.5, 1, or 2 h with 100 μg/mL Cy5.5-labeled Phl-erda or Phl-erda@Ab, with PBS-only samples serving as controls. Following incubation, cells were stained with LysoTracker Green and DiI as described earlier, then detached with trypsin. After two washes in ice-cold PBS, they were resuspended in 500 μL PBS for flow cytometry analysis on a BD FACS Calibur. The resulting data were analyzed with FlowJo software (version 10.8.1).

#### 4.2.6. Receptor Blocking Assay

Cells were cultured until 80–90% confluence. Then, 1 mL of trypsin was added for 2 min of digestion, which was terminated by the addition of complete culture medium. Cells were centrifuged at 1000 rpm for 5 min and resuspended in 1 mL of medium. For confocal imaging, cells were plated in confocal dishes (1.5 × 10^5^ cells/dish) and allowed to adhere for 24 h. For the receptor blocking assay, to block *FGFR3*, cells were exposed to free *FGFR3* antibody (10 µL, 1.5mg/ml) for 1 h at 4 °C prior to liposome treatment, with 1 mL of sterile PBS used as the solvent control. After incubation, the medium containing the free antibody was removed, and cells were washed two to three times with pre-chilled PBS. Subsequently, cells were incubated with Phl-erda@Ab (100 µg/mL, Cy5.5-labeled liposomes) in serum-free medium at 37 °C for 1 h. In the control group, cells were treated without the free antibody, while all other procedures remained identical. After incubation, cells were washed with pre-chilled PBS and then non-internalized nanoparticles were removed by a glycine wash (0.2 M glycine, 0.15 M NaCl, pH 3.0; 5–10 min, on ice) that remained bound to the cell surface. Cells were then washed again with PBS. For confocal microscopy, cells were fixed with 1 mL of 4% paraformaldehyde at room temperature for 15–20 min. Images were acquired using a confocal microscope at an excitation wavelength of 561 nm (Cy5.5 channel). For flow cytometric quantification of intracellular fluorescence, the fixation step was omitted; instead, cells were directly detached with trypsin, collected, and resuspended in PBS.

Image analysis was performed using ImageJ/Fiji software. Regions of interest (ROIs) were manually outlined along the Cy5.5-labeled cell membrane contours. The integrated fluorescence intensity of erdafitinib was measured within each ROI, and background fluorescence was measured in cell-free areas adjacent to each ROI. The background-corrected fluorescence results are calculated using the following formula:

(5) CTCF=Integrated−(ROI Area×Mean Background Fluorescence)

The relative fluorescence intensity per cell was expressed as the CTCF value. Mean values for each group were obtained from all measured cells. All experiments were performed at least three times independently. For the receptor blocking assay, data were analyzed using two-way ANOVA (cell line and blockade as the two factors) followed by Tukey’s multiple comparisons test. For all other analyses, the statistical tests are specified in the corresponding figure legends. A *p*-value of <0.05 was considered statistically significant. Significance levels are indicated as follows: * *p* < 0.05, ** *p* < 0.01, *** *p* < 0.001, **** *p* < 0.0001.

#### 4.2.7. In Vitro Cytotoxicity Evaluation

Cell viability was assessed by CCK-8 assay to gauge how free erdafitinib, Phl, Phl-erda, and Phl-erda@Ab affected RT112 and RT4 cell proliferation. The protocol unfolded in three steps. First, cells were plated in 96-well plates at 5 × 10^3^ per well and left overnight to attach. Next, each formulation was applied at concentrations ranging from 0 to 640 μg/mL for 1 h; cells were then rinsed three times with PBS to clear unbound drug and switched to fresh medium for a 24 h recovery period. Finally, 10 μL CCK-8 reagent was added to each well, and plates were incubated for 1 h before reading absorbance at 450 nm on a SpectraMax i3x reader (Molecular Devices, LLC, (San Jose, CA, USA)).

#### 4.2.8. Live/Dead Viability Assessment

We used a live/dead fluorescence assay (calcein AM/PI, Beyotime Biotechnology (Shanghai, China)) to evaluate cell viability in RT112, RT4 and 5637 cultures after treatment, following the supplier’s guidelines. The procedure ran as follows. Cells were plated in 35 mm dishes and allowed to adhere overnight. The next day, medium was replaced with fresh preparations containing 100 μg/mL erdafitinib, Phl-erda, or Phl-erda@Ab; PBS alone served as the vehicle control. All exposures lasted 1 h. Afterward, cells were collected, rinsed twice with cold PBS, and resuspended in binding buffer. Fourth, the suspensions were stained with calcein AM and PI for 15 min in the dark. Finally, stained cells were visualized under a confocal microscope. Each treatment condition was carried out in triplicate.

### 4.3. In Vivo Experiments

#### 4.3.1. Animals and Ethical Compliance

The animal experiments were performed using female BALB/c nude mice obtained from a commercial supplier (Beijing Viton-Lihua Laboratory Animal Technology Co., Ltd.(Beijing, China)). The *mice* were 6–7 weeks old, weighed 18–22 g at the time of arrival, and were housed under specific pathogen-free conditions throughout the study. The housing environment was maintained at 22 ± 2 °C, with 50 ± 10% relative humidity and a 12 h light/dark cycle, and the animals had free access to sterilized food and water. All procedures were conducted in compliance with the NIH guidelines for laboratory animal care and the 3Rs principles. The research protocol received ethical clearance from the Animal Welfare and Ethics Committee of Fourth Military Medical University (approval No. IACUC-20250177).

#### 4.3.2. Orthotopic Bladder Tumor Implantation

An orthotopic model of bladder carcinoma was generated via intravesical administration of RT112 cells, preceded by chemical disruption of the bladder epithelial layer. The surgical procedure was performed under sodium pentobarbital anesthesia (100 mg/kg, intraperitoneal injection). A blunt 27-gauge needle, coated with glycerol for lubrication, was carefully inserted through the urethral opening into the bladder. The urethra was then rinsed with PBS three times. Next, 0.1 M HCl was instilled for 30 s, followed by a PBS wash; this was repeated with 0.1 M NaOH for another 30 s, with another PBS rinse in between. Finally, 50 μL RT112 cell suspension (2 × 10^7^ cells/mL in Matrigel) was delivered into the bladder. The urethral orifice was clamped with forceps and immediately ligated with a size-1 suture to prevent cell suspension leakage. After 1 h, the ligation was removed, and the mice were placed in clean cages for recovery.

#### 4.3.3. Intravesical Tumor Targeting and Retention Assessment

Tumor Targeting. The in vivo targeting ability of Phl-erda@Ab to *FGFR3*-positive bladder tumors was evaluated in orthotopic BCa-bearing nude mice (n = 3 per group). Mice received an intravesical instillation of 50 μL of FITC-labeled Phl-erda@Ab. After 1 h of urethral ligation, the bladders were harvested, sectioned, and co-stained with a red fluorescently labeled *FGFR3* antibody. Fluorescence co-localization was observed under a confocal microscope, and all procedures were conducted under dark conditions.

Intravesical Drug Retention. To assess the intravesical residence time of Phl-erda@Ab in the bladder, orthotopic BCa mice were randomly divided into two groups (*n* = 9 per group), including the Phl-erda group and the Phl-erda@Ab group (with erdafitinib labeled with Cy5.5, dose 4.81 mg/kg). After intravesical instillation of 50 μL of Phl-erda or Phl-erda@Ab, the urethra was clamped for 1 h, followed by free voiding. Baseline fluorescence signals were acquired by an IVIS Lumina XRMS III imaging system (PerkinElmer, Inc. (Waltham, MA, USA)) in the Cy5.5 channel immediately after instillation. Dynamic imaging was performed from 1 to 8 h post-instillation, and fluorescence intensities were quantitatively analyzed to calculate the drug retention rate in the bladder relative to the 1 h baseline.

#### 4.3.4. Therapeutic Efficacy Evaluation

We tested the therapeutic potential of each formulation in an orthotopic bladder cancer model. Tumor-bearing mice were divided at random into four groups (*n* = 3) and treated intravesically with either PBS, free erdafitinib, Phl-erda, or Phl-erda@Ab. Before every instillation, the bladder was emptied by gentle compression and washed with PBS. Each mouse received 50 μL formulation at an erdafitinib-equivalent dose of 4.81 mg/kg; the urethra was then clamped for 1 h to promote drug retention. Treatments were given every 4 days for 28 days total. Bioluminescence was monitored weekly on an IVIS Lumina XRMS III (PerkinElmer, Inc. (Waltham, MA, USA)). For each animal, tumor burden was gauged by normalizing the post-treatment signal against its own pre-treatment baseline.

#### 4.3.5. Histology and Immunohistochemistry

After the last treatment, mice were euthanized and their bladders removed for histological analysis. For H&E staining, sections were first immersed in hematoxylin for 2 min, then rinsed under running water and placed in eosin for another 2 min. They were subsequently dehydrated through an alcohol gradient, cleared in xylene, and mounted with neutral resin.

Ki-67 immunohistochemistry began with dewaxing and rehydration, followed by antigen retrieval in EDTA (pH 9.0) for 15 min using heat. Endogenous peroxidase was quenched with 3% H_2_O_2_, and nonspecific binding was blocked with serum. Slides were incubated with primary anti-Ki-67 antibody overnight at 4 °C, then treated with HRP-conjugated secondary antibody for 30 min at room temperature. DAB was used for color development, with hematoxylin as a counterstain.

For TUNEL staining, sections underwent Proteinase K digestion and Triton X-100 permeabilization, followed by H_2_O_2_ quenching and serum blocking. The TUNEL reaction mix was then applied and incubated at 37 °C for 1 h in the dark, after which nuclei were stained with DAPI.

#### 4.3.6. Organ Toxicity Evaluation

At sacrifice, the heart, liver, spleen, lungs, and kidneys were harvested and fixed for H&E histology. Staining entailed hematoxylin (2 min), water rinse, eosin (2 min), ethanol dehydration, xylene clearing, and resin mounting.

### 4.4. Statistical Processing

GraphPad Prism 9.0 (GraphPad Software (San Diego, CA, USA)) was employed for all statistical evaluations. Data are expressed as mean ± standard deviation (SD) unless otherwise specified. The normality of data distribution was assessed using the Shapiro–Wilk test. Comparisons between two groups were analyzed using an unpaired two-tailed Student’s *t*-test. For multi-group comparisons, one-way or two-way analysis of variance (ANOVA) followed by Tukey’s post-hoc multiple comparison test was applied. A two-sided *p*-value < 0.05 was considered statistically significant. The number of biological replicates (n) is indicated in the corresponding figure legends.

## 5. Conclusions

In this work, we developed Phl-erda@Ab—liposomes loaded with erdafitinib that release their cargo in response to acidic pH (via hydrazone bond cleavage) and carry surface anti-*FGFR3* antibodies for intravesical delivery. Drug release was sharply pH-dependent, with preferential liberation at pH 5.3–6.5, while the antibody decoration enabled selective binding to *FGFR3*-overexpressing bladder cancer cells. In RT112 cultures and the corresponding orthotopic model, the targeted formulation outperformed its non-targeted counterpart on both retention and antitumor efficacy. Mechanistically, this translated into lower Ki-67 proliferation indices and higher TUNEL apoptotic positivity. Notably, repeated intravesical instillations produced no detectable toxicity in major organs—heart, liver, spleen, lung, or kidney. Taken together, our results offer preclinical support for advancing *FGFR3*-targeted intravesical nanotherapy toward the clinic—specifically as a strategy that might spare the bladder in patients whose tumors overexpress this receptor.

## Data Availability

The data that support the findings of this study are available from the corresponding author upon reasonable request.

## Supplementary Materials

The following supporting information can be downloaded at https://www.mdpi.com/article/10.3390/ijms27209014/s1.
