# Supplementary material for: pH-Responsive Erdafitinib Liposomes with FGFR3 Targeting for Intravesical Bladder Cancer Therapy
Source: Int J Mol Sci. 2026 Oct 11;27(20):9014. doi: 10.3390/ijms27209014 (PMC13704757; doi:10.3390/ijms27209014)
Supplement: Supplementary file 1 [file ijms-27-09014-s001.zip › ijms-4464996-supplementary.pdf]

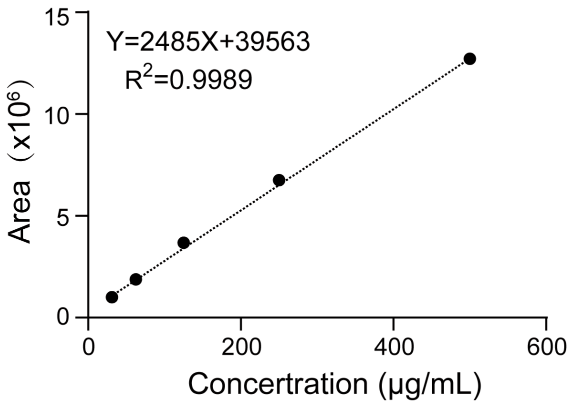

Figure S1. Calibration curve of erdafitinib in PBS solution. The standard curve exhibited an excellent goodness of fit ($R^2 > 0.999$).

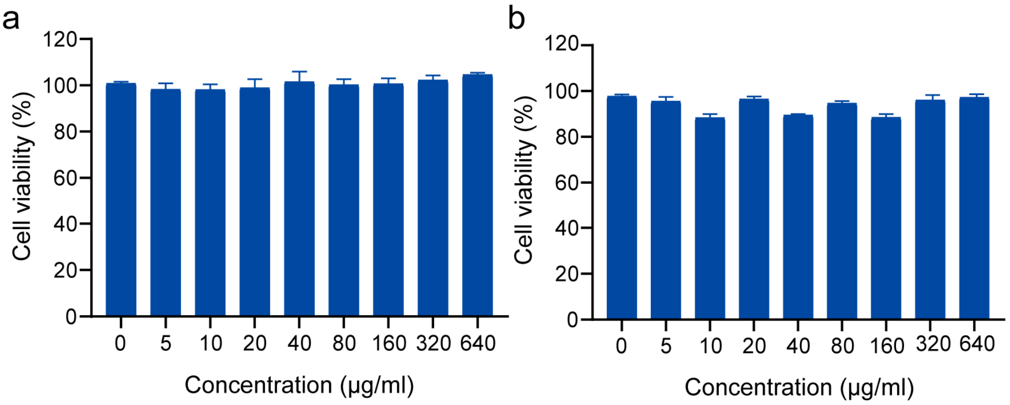

Figure S2. Effect of Phl on the viability of (a) RT112 and (b) 5637 cells at different concentrations. Regardless of the concentration, cell viability in both RT112 and 5637 cells remained above 80% after treatment with Phl, indicating good biocompatibility and supporting its use as a safe drug carrier.

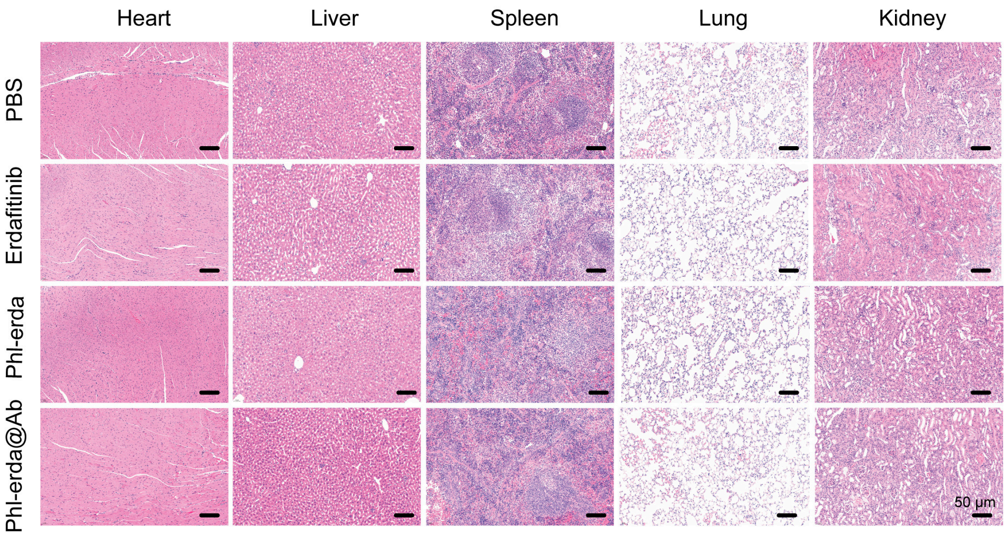

Figure S3. Representative H&E staining images of the heart, liver, spleen, lung, and kidney from nude mice in each group. No obvious tissue damage or pathological alterations were observed in any of the major organs after treatment, indicating the favorable systemic safety of the intravesical instillation strategy. Scale bars: 50 μm.

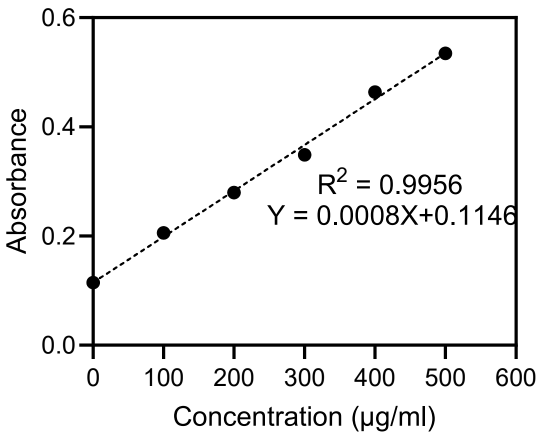

Figure S4. Calibration curve of BSA protein standards at various concentrations. The standard curve exhibited an excellent goodness of fit.

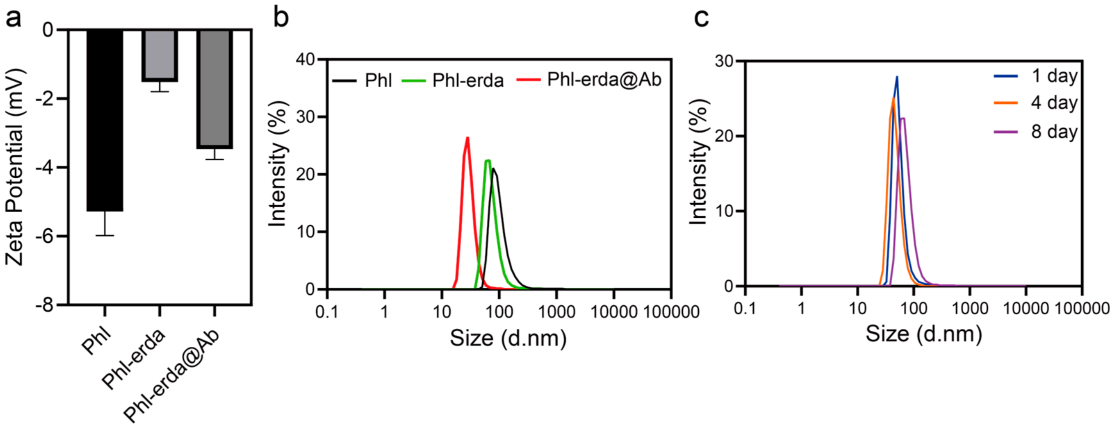

Figure S5. Characterization of Phl, Phl-erda, and Phl-erda@Ab in artificial urine. (a) Zeta potentials of Phl, Phl-erda, and Phl-erda@Ab in artificial urine. (b) The hydrodynamic diameters of Phl, Phl-erda, and Phl-erda@Ab were 274.1 nm, 231.6 nm, and 209.6 nm, respectively, with corresponding PDI values of 0.538, 0.522, and 0.934. (c) The hydrodynamic diameters of Phl-erda on days 1, 4, and 8 after synthesis were 231.6 nm, 221.6 nm, and 325 nm, respectively, with corresponding PDI values of 0.522, 0.610, and 0.548.

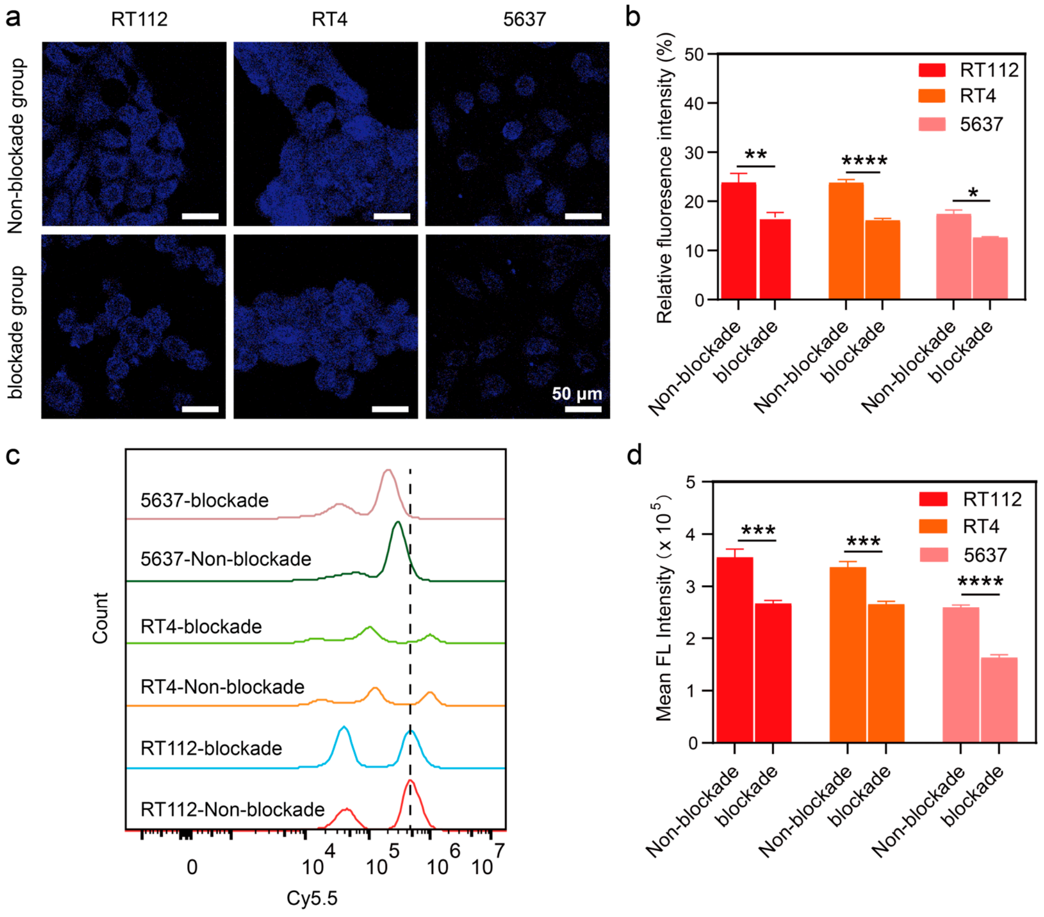

Figure S6. FGFR3 receptor blockade. (a) CLSM images of cellular uptake of CY5.5-labeled Phl-erda@Ab in RT112, RT4, and 5637 cells after 1 h of incubation in the blockade group and the non-

blockade group. Blue represents cy5.5. Scale bar: 50 μm. (b) Semiquantification of fluorescence intensity of Phl-erda@Ab in cells. (c) Representative flow cytometry histograms and (d) quantitative analysis of Phl-erda@Ab fluorescence in cells. In Figure S6c, the dashed lines on the y-axis represent the peak fluorescence intensity positions of different groups at various time points, using RT112-non-blockade as the reference for comparison. This approach allows for a direct visual comparison of fluorescence intensity values under different treatment conditions. Data were analyzed using two-way ANOVA followed by Tukey's multiple comparisons test. Significance levels are indicated as follows: *P < 0.05, **P < 0.01, ***P < 0.001, ****P < 0.0001.

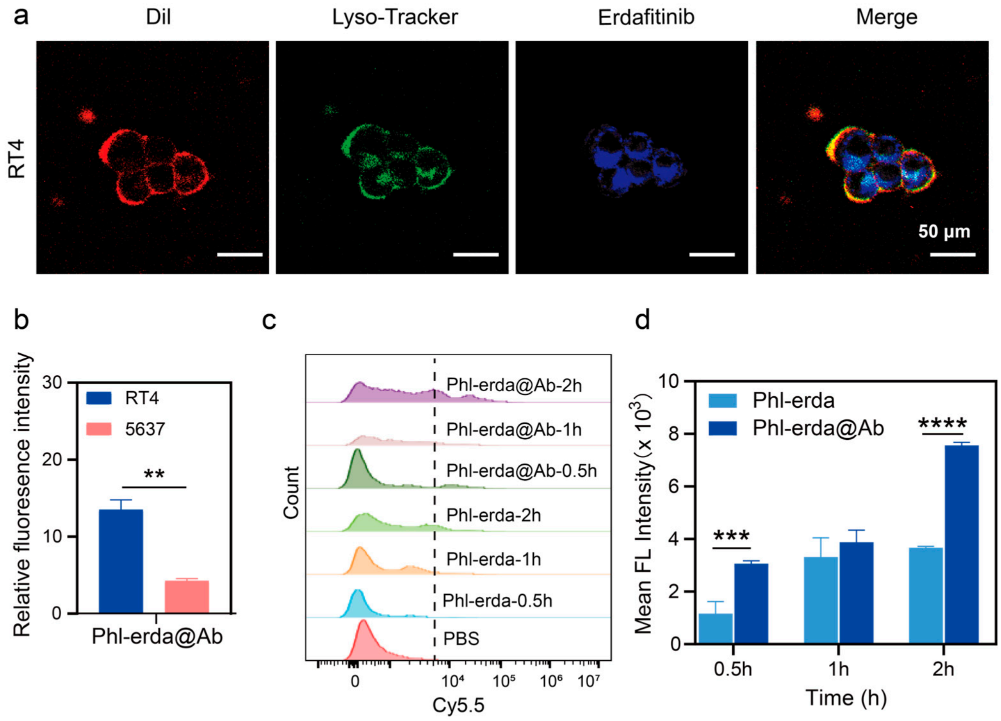

Figure S7. Cellular uptake of Phl-erda@Ab in RT4 cells. (a) CLSM images showing the cellular uptake of Phl-erda@Ab in RT4 cells after 1 h of incubation. The cell membrane, lysosomes, and Phl-erda@Ab were labeled with Dil (red), Lyso-Tracker (green), and Cy5.5 (blue), respectively. Scale bar: 50 μm. (b) Semi-quantitative analysis of intracellular Phl-erda@Ab fluorescence intensity. (c) Representative flow cytometry histograms and (d) quantitative analysis of Phl-erda@Ab fluorescence signals in cells. In Figure S7c, the dashed lines on the vertical axis indicate the positions of peak fluorescence intensity among different groups at various time points, with Phl-erda@Ab-2h used as the reference for comparison. This method allows direct comparison of differences in fluorescence intensity values under different treatment conditions. Data were analyzed by one-way ANOVA. Significance levels are indicated as follows: **P < 0.01, ***P <

0.001, ****P < 0.0001.

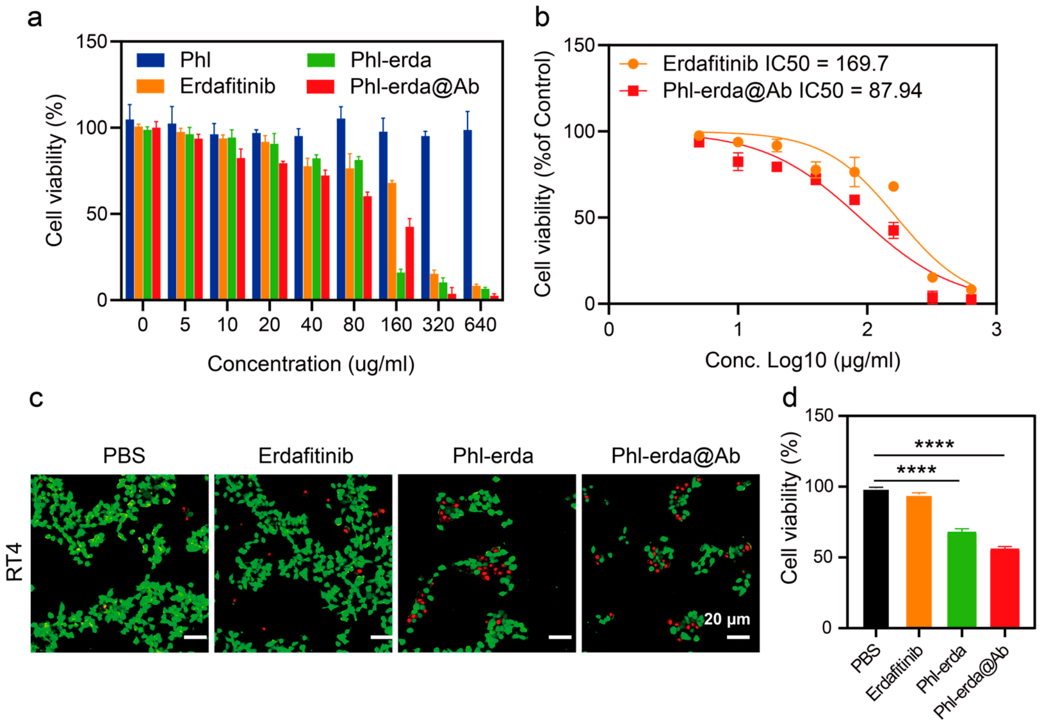

Figure S8. In vitro cytotoxicity of Phl-erda@Ab in RT4 cells. (a) Cytotoxic effects of Phl, erdafitinib, Phl-erda, and Phl-erda@Ab at different concentrations on RT4 cells. (b) IC50 values of erdafitinib and Phl-erda@Ab in RT4 cells. (c) CLSM images of RT4 cells stained with Calcein AM (live cells, green) and PI (dead cells, red) after 24 h of incubation in different treatment groups. Scale bar: 20 µm. (d) Relative viability of RT4 cells after live/dead staining. Data were analyzed by one-way ANOVA. Significance levels are indicated as follows: ****P < 0.0001.

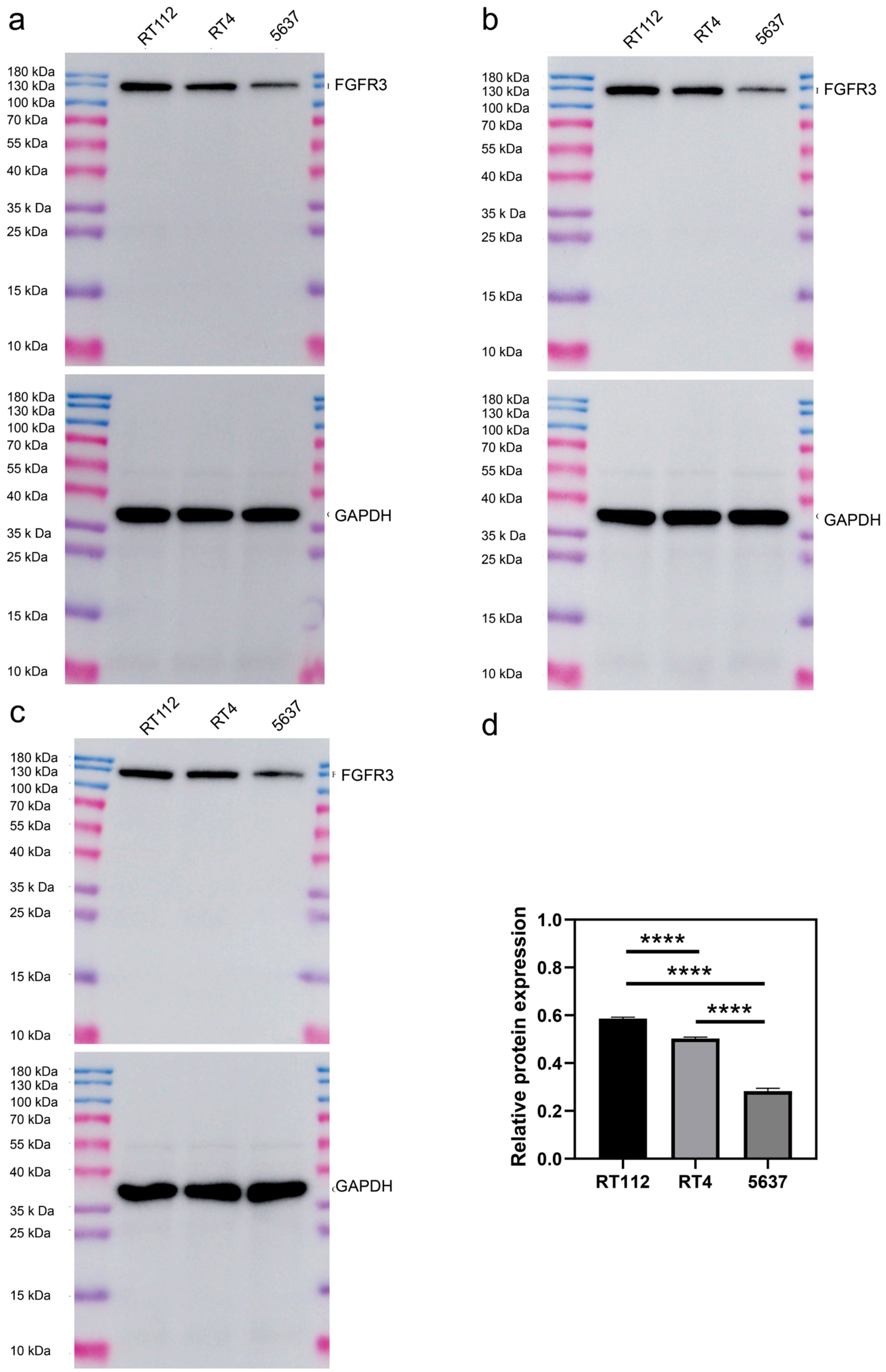

Figure S9. Expression and quantification of FGFR3 protein in cells. (a-c) FGFR3 protein expression in RT112, RT4, and 5637 cells was detected by Western blotting, with GAPDH as the loading control. The results are from three independent experiments. (d) Quantitative analysis and statistical

comparison of the results from three independent experiments. Data were analyzed using one-way ANOVA. Significance levels are indicated as follows: ****P < 0.0001.
